# Perturbation of the gut microbiome by *Prevotella spp*. enhances host susceptibility to mucosal inflammation

**DOI:** 10.1038/s41385-020-0296-4

**Published:** 2020-05-20

**Authors:** Aida Iljazovic, Urmi Roy, Eric J. C. Gálvez, Till R. Lesker, Bei Zhao, Achim Gronow, Lena Amend, Sabine E. Will, Julia D. Hofmann, Marina C. Pils, Kerstin Schmidt-Hohagen, Meina Neumann-Schaal, Till Strowig

**Affiliations:** 1 Department of Microbial Immune Regulation, Helmholtz Center for Infection Research, Braunschweig, Germany; 2 Hannover Medical School, Hannover, Germany; 3 Bacterial Metabolomics, Leibniz institute DSMZ-German Collection of Microorganisms and Cell Cultures, Braunschweig, Germany; 4 Department of Bioinformatics and Biochemistry, BRICS, Technische Universität Braunschweig, Braunschweig, Germany; 5 Mouse Pathology, Helmholtz Center for Infection Research, Braunschweig, Germany; 6 Centre for Individualised Infection Medicine, Hannover, Germany

## Abstract

Diverse microbial signatures within the intestinal microbiota have been associated with intestinal and systemic inflammatory diseases, but whether these candidate microbes actively modulate host phenotypes or passively expand within the altered microbial ecosystem is frequently not known. Here we demonstrate that colonization of mice with a member of the genus *Prevotella*, which has been previously associated to colitis in mice, exacerbates intestinal inflammation. Our analysis revealed that *Prevotella intestinalis* alters composition and function of the ecosystem resulting in a reduction of short-chain fatty acids, specifically acetate, and consequently a decrease in intestinal IL-18 levels during steady state. Supplementation of IL-18 to *Prevotella*-colonized mice was sufficient to reduce intestinal inflammation. Hence, we conclude that intestinal *Prevotella* colonization results in metabolic changes in the microbiota, which reduce IL-18 production and consequently exacerbate intestinal inflammation, and potential systemic autoimmunity.

## Introduction

Intestinal homeostasis is maintained by the dynamic interplay between the gut microbiota and the host immune system,^1^ in which multiple cell types including intestinal epithelial cells (IECs) and goblet cells, serve not only as a passive barrier but also as a source of antimicrobial substances strengthening the barrier.^2^ Microbiota-derived metabolites represent important signals that impact both the mucosal immune system and proper epithelial barrier function.^3^ Alterations in the composition and function of the microbiota have been associated with a wide range of human disease including inflammatory bowel disease (IBD) and rheumatoid arthritis (RA). In IBD, it has been specifically hypothesized that immune-mediated pathologies arise from dysregulated immune responses towards the intestinal microbiota,^4,5^ but different other non-exclusive concepts about how the microbiota promotes IBD and potentially other autoimmune diseases are debated.^6^ For instance, an overall loss of microbial diversity, changes in the balance between beneficial commensals and potential pathobionts as well as changes in microbial metabolites such as short-chain fatty acids (SCFAs) have been reported in diverse patient populations.^7–9^ Strikingly, altered SCFA production also modulates systemic immune responses linking intestinal dysbiosis and extra-intestinal immunity.^10^ Still, the exact identity of intestinal bacteria and their metabolites that trigger aberrant host responses and contribute to the development of IBD and other autoimmune diseases in humans are not exactly known, as the direct causal relationship between microbiota and complex diseases has been difficult to prove outside animal models. For instance, several studies in humans described associations between IBD and increased abundance in Gammaproteobacteria and the presence of Enterobacteriaceae, particularly adherent-invasive *E. coli* (AIEC) strains.^11^ Notably, AIEC modulate colitis susceptibility in some mouse models^12,13^ and additional members of the Enterobacteriaceae family, i.e., *Klebsiella pneumoniae* and *Proteus mirabilis* were also identified to promote colitis in mice.^14,15^ Moreover, several other members of the murine microbiota were identified to directly exacerbate intestinal inflammation. This includes *Akkermansia muciniphila*^16^ as well as distinct *Bacteroides*^17^ and *Helicobacter* species.^18^ Of note, recent studies have also started to shed light on the role of non-bacterial members of the microbiome such as protozoa and phages in the development of IBD, i.e., the increased intestinal inflammation in mice colonized with *Tritrichomonas muris*,^19^ and an enrichment of Caudovirales bacteriophages in IBD patients.^20^

Beyond these well-studied examples, microbiome studies have identified many microbes that were found enriched in disease-promoting communities, but with unknown roles in host-microbiota crosstalk, i.e., members of the *Prevotella* genus.^21,22^ In general, the role of members of the *Prevotella* genus within the intestinal microbiota and their effects on the host is not completely understood and somewhat conflicting interpretations have been reported. High prevalence and relative abundance of *Prevotella* is found in non-Westerners who consume a plant-rich diet.^23,24^ Moreover, it has been shown that *Prevotella* spp. can improve glucose metabolism stimulated by the intake of prebiotics.^25^ Together, these studies suggest that *Prevotella* spp. are beneficial microbes that have colonized humans for extended periods of time. In contrast, other studies have associated *Prevotella* spp. with autoimmune diseases, insulin resistance and diabetes, and gut inflammation.^22,26,27^ Specifically, an overabundance of *Prevotella copri* was noted in new-onset rheumatoid arthritis (NORA) patients^22^ and also in patients with systemic autoimmunity associated with RA, but without clinical symptoms yet.^28^ In mouse models, an altered gut microbiota dominated by a member of the genus *Prevotella* was discovered in NLRP6-deficient mice and was associated with higher susceptibility to chemically-induced colitis.^21^ Interestingly, *Prevotella* spp. along with segmented filamentous bacteria (SFB) and *Helicobacter* spp. are among the highest immunoglobulin (Ig) A-coated bacteria in these mice, which has been interpreted to reflect their immunogenic features.^29^ These seemingly opposing effects by *Prevotella* on the host’s physiology may be caused by multiple factors including direct or community-mediated effects. Moreover, these effects may not be causally linked to the presence of *Prevotella* and other members of the *Prevotella*-dominated microbiome may have the propensity to promote inflammation and intestinal dysbiosis. However, the detailed investigation of the immunomodulatory properties of *Prevotella* spp. and their potential mechanisms is prohibited by the strain diversity, as well as the lacking availability of diverse intestinal *Prevotella* isolates from model organisms such as the mouse. As of now, culture collections include several *Prevotella* species from the human intestine, while no species isolated from mice are available.

In the present study, we isolated a novel intestinal *Prevotella* species (*Prevotella intestinalis* nov. sp.) from the colitogenic microbiota of *Nlrp6*^*−/*−^ mice and then investigated the impact of its colonization on the interplay between host and the microbiota during intestinal homeostasis and inflammation. We found that *P. intestinalis* colonization of WT specific pathogen-free (SPF) mice, devoid of any *Prevotella* spp. in the intestine, reshapes the resident intestinal microbial community and significantly alters the metabolic profile in the intestine. *Prevotella*-induced decrease in the levels of SCFA, in particular acetate, is associated with reduced colonic IL-18 expression and production during homeostasis. Notably, *P. intestinalis*-induced decrease of IL-18 production modulates the exacerbation of colonic inflammation in immunocompetent mice. Strikingly, colonization of a distinct SPF mouse line with *P. intestinalis* phenocopied the alterations including the enhanced susceptibility to DSS-induced intestinal inflammation demonstrating the effect can be observed in diverse microbial ecosystems. Colonization of SPF mice with another *Prevotella* spp. or a member of the Muribaculaceae family, which are both able to establish high relative abundance in SPF mice thereby disturbing the microbial ecosystem similarly reduce acetate production and affect intestinal inflammation. Together, our results firmly establish that a representative member of the genus *Prevotella*, namely *Prevotella intestinalis*, can causally promote inflammation in the host, an effect it shares with other gut bacteria. Enhancement of disease severity is associated to changes in the microbial ecosystem and microbially-produced metabolites.

## Results

### A novel *Prevotella* species*, Prevotella intestinalis*, colonizes SPF WT mice in high abundance and reshapes the intestinal microbial community structure

**Fig. 1:**
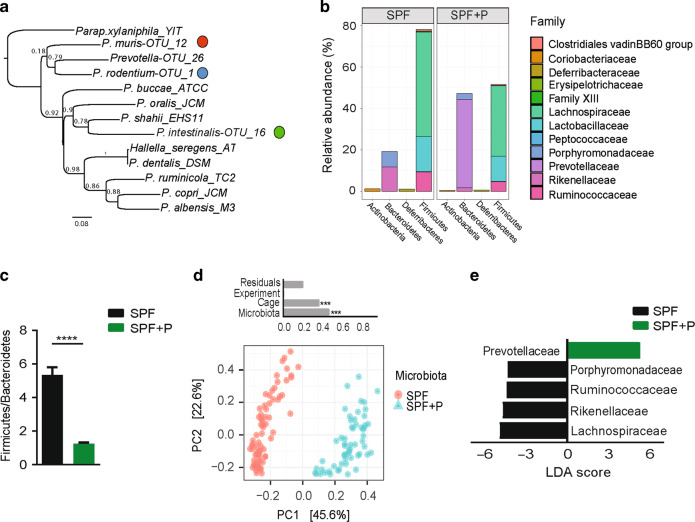
*P. intestinalis* colonization reshapes the resident SPF community. **a** Phylogenetic tree based on 16S rRNA sequences showing the positions of novel *Prevotella* species within the genus *Prevotella*. **b** Fecal microbiota composition analysis of untreated (SPF) and *P. intestinalis*-colonized (SPF + P) WT mice on the family level using 16S rRNA gene sequencing. **c** Ratio of relative abundances between Firmicutes to Bacteroidetes (F/B). **d** Analysis of β-diversity (PCoA) using Bray–Curtis distances along with multivariate analysis of variance (ADONIS test) of variables ‘microbiota’, ‘experiment’, and ‘cage’. **e** Analysis of differentially abundant bacterial families in SPF and SPF + P mice by LEfSe (Kruskal–Wallis test *p* < 0.05, LDA 4.0). Data in (**b**–**f**) represent *n* = 58–65 mice/group as mean ± SEM from ten independent experiments. *P* values represent an unpaired nonparametric Mann–Whitney test if not indicated differently. *p < 0.05; **p < 0.01; ***p < 0.001; ****p < 0.0001.

Alteration in the microbiota of some lines of *Nlrp6*^*−/−*^ mice renders them more susceptible to chemically-induced intestinal inflammation^30,31^ and a high relative abundance of unknown members of the family Prevotellaceae was identified by 16S rRNA gene sequencing in mice with high disease susceptibility.^21^ In addition, we identified that different species of the genus *Prevotella* were also highly abundant in other colitogenic communities.^32^ In order to experimentally address whether *Prevotella* spp. modulate disease severity, we isolated novel *Prevotella* species from the colon content of these mouse lines using a step-wise enrichment and isolation scheme under strictly anaerobic conditions. This cultivation effort yielded three new species belonging to the genus *Prevotella* based on the comparison of their 16S rRNA genes to other described *Prevotella* species (Fig. 1a). Based on genotypic characterization we conclude that each of the isolates belongs to a novel bacterial taxon within the genus *Prevotella*, for OTU_16 isolated from *Nlrp6*^*−/−*^ mice the name *Prevotella intestinalis* is proposed (Table S1) (Gálvez, Iljazovic, manuscript in preparation). Of note, mining of metagenomic sequencing data from the murine gut using a recently released resource, the integrated mouse gut metagenome catalog (iMGMC),^33^ revealed that *P. intestinalis* can be found in both laboratory and wild mice demonstrating it is a naturally occurring bacteria in the mouse gut (Table S2).

To study the impact of *P. intestinalis* on the intestinal ecosystem, we colonized specific pathogen-free (SPF) WT mice, devoid of any *Prevotella* species, by a single oral gavage (SPF + P). After 4–5 weeks, *P. intestinalis* colonization was determined by analyzing fecal microbiota composition using 16S rRNA gene sequencing. Strikingly, *P.* *intestinalis* colonized SPF + P mice in high relative abundance (42.5% +/− 2.9, mean +/− SEM) (Fig. 1b), thereby significantly reshaping the microbial community including a decreased Firmicutes to Bacteroidetes ratio (F/B) (Fig. 1c). In addition, analysis of β-diversity using principle coordinates analysis (PCoA) showed distinct clustering of SPF and SPF + P communities (Fig. 1d). Based on permutational multivariant analysis of variance (ADONIS), over 45% of the differences were attributed to *Prevotella* colonization (*R*^2^ = 0.46, *p* < 0.001). Although there was no difference in observed species richness (*p* = 0.26), the complexity of the community structure, when accounting for species richness and evenness (Shannon index), was significantly lower in SPF mice after *P. intestinalis* colonization (*p* < 0.0001) (Supplementary Fig. 1a). On a family level, comparison of SPF communities with and without *P. intestinalis* colonization by linear discriminant analysis (LDA) effect size (LEfSe) showed that *Prevotella* colonization decreased relative abundance of resident families within Bacteroidetes phyla, as well as the predominant Firmicutes, namely Lachnospiraceae and Ruminococcaceae (Fig. 1e).

*Prevotella* spp. have been found to predominantly colonize the lumen of the lower gastrointestinal tract (GIT),^30,34^ but have been as well described as a part of the intestinal mucosal community.^35–37^ The combination of 16S rRNA gene sequencing with the quantitation of microbial loads has recently described variation within the absolute abundances of intestinal bacteria and linked it to enterotypes in healthy humans.^38^ Hence, we quantified bacterial loads using flow cytometry-based enumeration of bacterial concentrations in the luminal content (Supplementary Fig. 1b). This revealed no differences in the total bacterial cell counts after *Prevotella* colonization suggesting that *Prevotella* is not simply increasing the total microbial density, but rather replaces other bacteria (Supplementary Fig. 1c). We additionally analyzed the composition of the mucosa-associated microbiota in distal and proximal colon (DC and PC), locations with highest *P. intestinalis* colonization. We found *P. intestinalis* to be present in both DC and PC mucosal sites, with higher abundance in the DC (23.9% +/− 2.6, mean +/− SEM), yet significantly lower than in the DC lumen (Supplementary Fig. 1d). No translocation into the colonic tissue, mesenteric lymph nodes or the liver were detected (Supplementary Fig. 1e). Altogether, this data demonstrate that *P. intestinalis* colonization has a significant impact on SPF community structure, including the decrease in the microbial diversity and Firmicutes to Bacteroidetes ratio. In addition, *P. intestinalis* predominantly colonizes the lumen of the colon, however, it is also found closely associated to the colonic mucosa, which is in line with previous findings regarding the niche of *Prevotella* spp. and where it may exert immunomodulatory effects on the host.^39^

### *Prevotella intestinalis* colonization is sufficient to exacerbate DSS-induced colitis in immunocompetent host

**Fig. 2:**
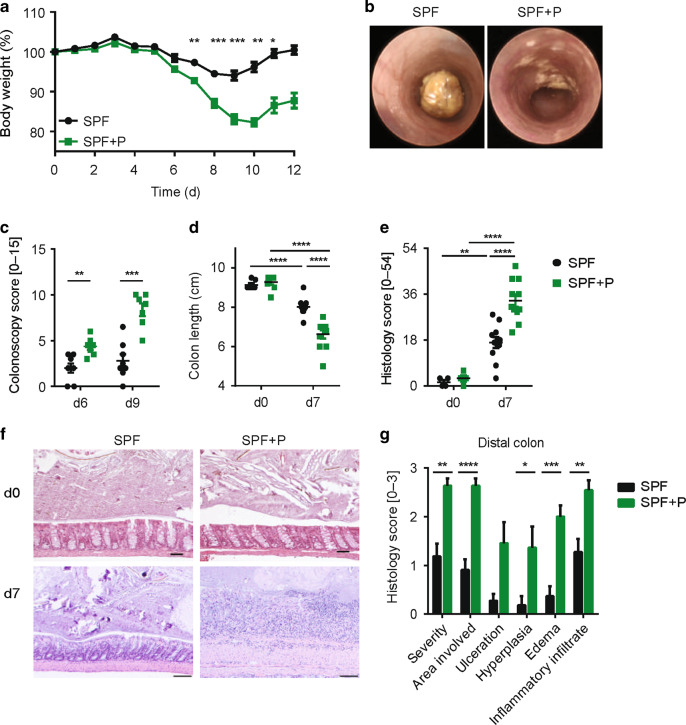
*P. intestinalis* colonization alters the colitis susceptibility of WT SPF mice. **a** Body weight loss of WT mice harboring SPF and SPF + P microbiota during DSS colitis (2.1% w/v for 7 days). **b** Representative colonoscopy images of colitis severity performed on day 6 after colitis induction in SPF and SPF + P mice. **c** Colitis score on day 6 and day 9 of DSS colitis, based on five parameters: granularity of mucosal surface, vascular pattern, translucency of the colon mucosa, visible fibrin, and stool consistency. **d** Colon length and **e** histological evaluation of SPF and SPF + P mice measured during steady state (d0) and during DSS colitis (d7). **f** Representative images of H&E-stained distal colon sections of d0 and d7 of DSS colitis and **g** histological analysis of d7 distal colon sections by each scoring parameter. Results represent *n* = 4–8 mice/group as mean ± SEM from one out of three representative experiments. *P* values represent an unpaired nonparametric Mann–Whitney test. *p* < 0.05; **p < 0.01; ***p < 0.001; ****p < 0.0001.

We next investigated whether *P. intestinalis* can exacerbate susceptibility to intestinal inflammation after induced damage to the intestinal barrier. Therefore, acute intestinal inflammation was induced in littermate SPF and SPF + P mice by administering dextran sulfate sodium (DSS) in drinking water (2.1% w/v). While WT SPF mice used in this study have been previously reported to be relatively resistant to induction of DSS colitis, displaying moderate colitis severity and mild weight loss,^32^ colonization of SPF + P mice resulted in a more severe disease outcome such as significant increased body weight loss (Fig. 2a). Of note, SPF + P mice did not show an increase in mortality (Supplementary Fig. 2a). Colonoscopy on days 6 and 9 after induction of DSS colitis revealed increased tissue damage in *P.* *intestinalis*-colonized mice (Fig. 2b, c). Moreover, higher intestinal inflammation in SPF + P mice was supported by pronounced colon shortening (Fig. 2d) and histological characterization of tissue damage during DSS colitis (Fig. 2e, f). Specifically, inflammation in SPF + P mice was highest in the distal colon with pronounced tissue erosion, and higher hyperplasia, edema, and infiltration of inflammatory cells (Fig. 2g). Histological analysis of cecum and small intestine during DSS colitis showed no significant differences between SPF and SPF + P mice (Supplementary Fig. 2b, c). Microbiota analysis demonstrated that the relative abundance *of P. intestinalis* decreased during DSS colitis suggesting that it cannot benefit from the altered milieu during inflammation (Supplementary Fig. 2d). While damage of the intestinal barrier has been demonstrated to result in the translocation of some groups of intestinal bacteria, no translocation of *P. intestinalis* was detected using a in intestinal tissue, mLN nor the liver (Supplementary Fig. 2e).

Microbiota composition and functionality has the potential to influence the outcome of many types of animal models of diseases.^40^ Whether the enhancement of intestinal inflammation by *P. intestinalis* was only specific to the SPF mice raised in our facility, SPF mice devoid of *P. intestinalis* were obtained from a commercial vendor (Taconic, referred to as SPF2). SPF2 were colonized by a distinct *Prevotella* species that was present in lower abundance (4.9% +/– 1.0, mean +/− SEM), but *P. intestinalis* outcompeted this species and further expanded within the SPF2 mice (SPF2 + P) (13.0% +/− 2.4) (Supplementary Fig. 2f, g). After induction of DSS colitis SPF2 + P displayed higher body weight loss, increased reduction of colon length and higher mortality compared with SPF2 mice demonstrating that *P. intestinalis*-enhanced intestinal inflammation is not limited to a specific SPF mouse line (Supplementary Fig. 2h-l). Together, these data establish that *P. intestinalis* is able to alter susceptibility to DSS colitis in an immunocompetent host.

### Altered DSS susceptibility by *Prevotella intestinalis* colonization is associated with elevated pro-inflammatory cytokine responses and is independent of adaptive immunity

**Fig. 3:**
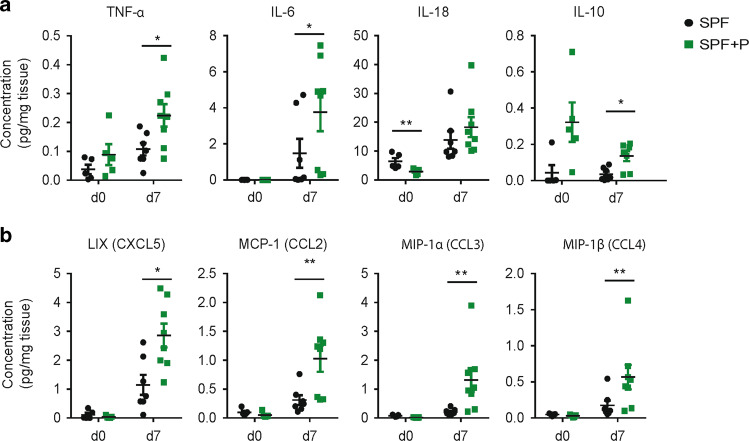
*Prevotella*-exacerbated intestinal inflammation is characterized by increased levels pro-inflammatory cytokines and chemokines. **a** Cytokines and **b** chemokines measured from distal colon tissue homogenates of SPF and SPF + P mice on day 0 and day 7 of DSS, analyzed using LEGENDplex kit or ELISA (IL-18). Data represent *n* = 4–8 mice/group as mean ± SEM from one out of two representative experiments. *P* values indicated represent an unpaired nonparametric Mann–Whitney test. *p < 0.05; **p < 0.01; ***p < 0.001; ****p < 0.0001.

To characterize the differences in inflammation between SPF and SPF + P mice, various cytokines and chemokines were quantified in the distal colon tissue in steady state and during inflammation. Levels of the pro-inflammatory cytokines IL-6 and tumor necrosis factor alpha (TNF-α) were higher in mice harboring *P. intestinalis* (Fig. 3a) on day 7 of DSS colitis. *P. intestinalis* colonization also resulted in increased levels of the anti-inflammatory cytokine IL-10 in the colon. Notably, contrary to the results observed in SPF mice colonized with the *Prevotella*-rich microbial community from *Nlrp6*^*−/−*^ mice, from which *P. intestinalis* was originally isolated, SPF + P mice did not display increased levels of interferon γ (IFN-γ), IL-17A, IL-1β, or CCL5 (Supplementary Fig. 3).^32^ Strikingly, during steady state there was no impact of *P. intestinalis* colonization on the production of a range of tested cytokines such as IL-6, TNF-α, IL-10, IFN- γ, IL-17a, or IL-1β (Supplementary Fig. 3), when compared to the SPF mice, except a 2.5-fold decrease of IL-18 levels in distal colons of *Prevotella*-colonized mice. Higher intestinal inflammation in SPF + P mice during DSS colitis was also characterized by significant increases of multiple chemokines, including LIX and MCP-1, which have been involved in the recruitment and activation of monocyte and neutrophils to the site of inflammation, as well as MIP-1α and MIP-1β (Fig. 3b and Supplementary Fig. 3).

**Fig. 4:**
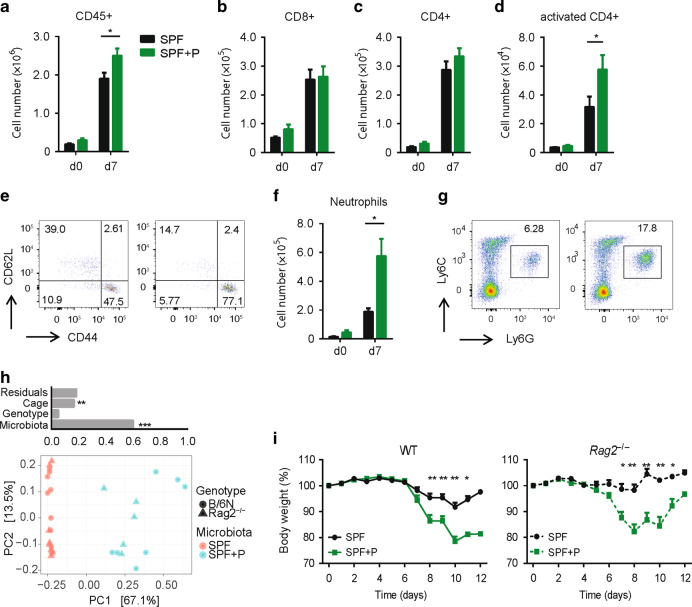
Inflammation in *Prevotella*-colonized mice is independent of adaptive immunity and associated with enhanced neutrophil recruitment. **a**–**g** Colonic lamina propria leukocytes (cLPLs) were isolated from WT mice harboring SPF and SPF + P microbiota, during the steady state (d0) and on day 7 (d7) during DSS colitis, and analyzed by fluorescence-activated cell sorting (FACS). **a** Total number of CD45+ leukocytes, **b** cytotoxic (CD8) T, **c** T helper (CD4), and **d** activated CD4 T cells in cLPLs with **e** representative FACS plots. **f** Total numbers of neutrophils on d0 and d7 DSS with **g** representative FACS plots of analysis of neutrophil infiltration upon DSS induction (d7). **h**–**i** WT and *Rag2*^*−/*−^ mice with SPF microbiota were left untreated or colonized with *P. intestinalis* for 5 week before induction of DSS colitis. **h** Analysis of β-diversity (PCoA) of WT and *Rag2*^*−/*−^ harboring SPF and SPF + P mice along with multivariate analysis of variance (ADONIS test) of variables ‘microbiota’, ‘genotype’, and ‘cage’. **i** Body weight of mice from Fig. 4h during DSS colitis (2.1% in drinking water for 7 days). Data represent *n* = 5–6 mice/group as mean ± SEM from one out of three representative experiments. *P* values indicated represent an unpaired nonparametric Mann–Whitney test unless stated otherwise. *p < 0.05; **p < 0.01; ***p < 0.001; ****p < 0.0001.

Inflammation in DSS colitis can be triggered by different effector cells including innate and adaptive immune cells.^32,41^ To identify which subsets of immune cells are differently presented between the two groups, we analyzed the abundance and composition of colonic lamina propria leukocytes (LPLs) before and 7 days after induction of DSS colitis by flow cytometry. *Prevotella* colonization did not result in increased numbers of LPLs (CD45+ cells) in the steady state, but resulted in increased numbers of LPLs after the DSS induction (Fig. 4a, Supplementary Fig. 4b). The global analysis of immune cells subsets of the adaptive immune system (Supplementary Fig. 4a) in colon tissue demonstrated no significant differences in cell numbers or frequencies, i.e., we observed no differences in the numbers and abundances of total CD4+ and CD8+ T cells (Fig. 4b, c) as well as B220+ B cells (not shown). Notably, while the numbers and frequency of activated CD4+ T cells (CD62L−CD44+) were increased in colons of SPF + P mice during DSS colitis (Fig. 4d, e), the numbers of different CD4+ T helper (Th) subsets including Th1 (CD4+IFN-γ+) and Th17 (CD4+IL-17A+) cells as well as in regulatory T cells (CD4+Foxp3+) were not affected (Supplementary Fig. 4c). Analyzing the abundance of various subsets of innate immune cells (Supplementary Fig. 4d), we observed a significant increase in frequency and numbers of neutrophils (Ly6C+Ly6G+ cells) in colons of mice colonized with *P. intestinalis* during DSS colitis, but not in the steady state (Fig. 4f, g, Supplementary Fig. 4e). These findings are in line with increased levels of multiple neutrophil-attracting chemokines we measured in colons of SPF + P mice (Fig. 3b). While no significant increase of infiltrating monocytes (Ly6c+Ly6g−MHCII^low-mid^+CCR2+) or dendritic cells (Ly6g−Ly6c+MHCII^high^+CD11c+) was noted by numbers (Supplementary Fig. 4f) or frequencies (not shown), we noted increased numbers of resident macrophages (Ly6g−Ly6c−CD11b+F4/80+MHCII+CX3CR1+) in SPF + P mice during DSS colitis (Supplementary Fig. 4f). These data suggested that exacerbation of DSS colitis severity by *P. intestinalis* is associated with differential recruitment and activation of innate and to a lesser degree of adaptive immune cells, respectively.

We recently demonstrated that the colitogenic community of *Nlrp6*^*−/−*^ mice, which contains *P. intestinalis*, alters susceptibility to DSS colitis via modulation of adaptive immune cells, i.e., transfer of the community in *Rag2*^*−/−*^ mice was unable to exacerbate disease severity.^32^ To test whether *P. intestinalis* requires the presence of adaptive immune cells to alter colitis susceptibility, we colonized WT and Rag2-deficient mice with *P. intestinalis*. Importantly, both WT and *Rag2*^*−/−*^ mice harbored the same SPF microbiota before the *P. intestinalis* colonization.^30^ Specifically, the comparison of their fecal microbiota composition before induction of DSS colitis showed that the mice clustered together in relation to their microbial communities (SPF or SPF + P) (Fig. 4h). Multivariate analysis of variance using ADONIS showed microbiota contributed to the variability of the groups with 60% (*R*^2^ = 0.60, *p* = 0.001), while genotype contributing to the differences as little as 5% (*R*^2^ = 0.05, *p* = 0.007). Strikingly, *P. intestinalis* exacerbated DSS colitis severity both in WT and *Rag2*^*−/−*^ mice, as indicated by their weight loss (Fig. 4i) and colon shortening (Supplementary Fig. 4g). Taken together, we conclude that *P. intestinalis* colonization promotes intestinal inflammation upon damage to the intestinal barrier independent of adaptive immune cells.

### *P. intestinalis*-induced decrease of IL-18 modulates the exacerbation of colonic inflammation

Besides differences in cytokine and chemokine production during DSS colitis, we also observed that *P. intestinalis* colonization of SPF mice resulted in a decrease of IL-18 levels in colonic tissue before induction of intestinal inflammation (Fig. 3a). The role of IL-18 during DSS colitis has been controversially discussed, either suggested to play a role in promoting intestinal epithelial integrity and protection from acute experimental colitis,^42–44^ or to exacerbate intestinal inflammation due to impaired repair processes.^45,46^ This prompted us to investigate whether lower levels of colonic IL-18 may be linked to the *Prevotella*-enhanced susceptibility to colonic inflammation during DSS-induced colitis.

**Fig. 5:**
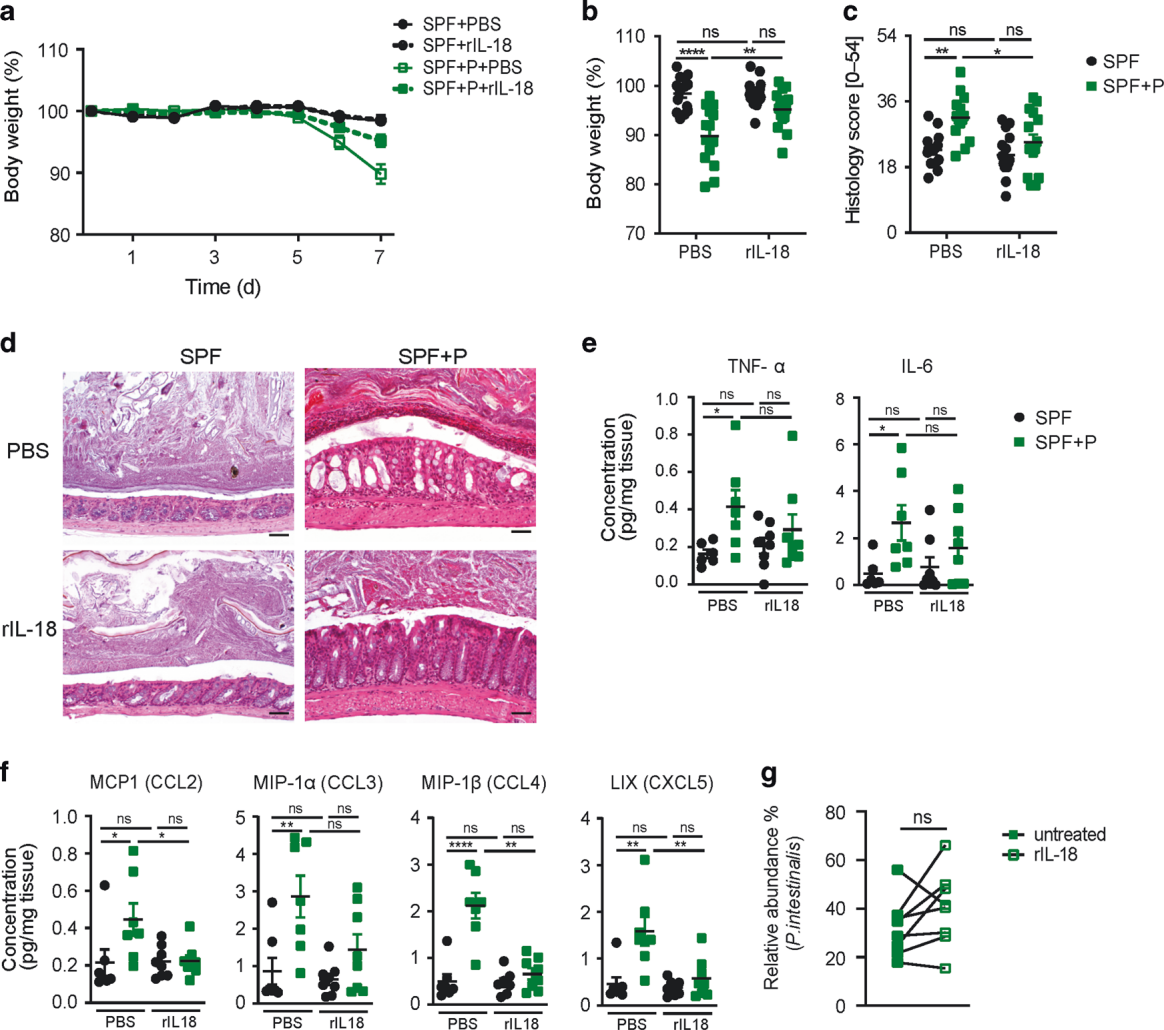
rIL-18 supplementation ameliorates *Prevotella*-induced exacerbation of colonic inflammation. **a** Body weight loss of SPF and SPF + P mice during DSS colitis, either treated with PBS or 200 ng rIL-18. **b** Percent body weight and **c** histological evaluation of colon tissue on day 7 of DSS colitis of mice from (**a**), with **d** representative images of H&E-stained distal colon sections on d7 of DSS colitis. **e** Cytokines and **f** chemokines measured from distal colon tissue homogenates of SPF and SPF + P mice (d7 DSS) treated with PBS or rIL-18, analyzed using LEGENDplex kit or ELISA. **g** Relative abundance of *P. intestinalis* in in SPF + P mice before (untreated) and after rIL-18 treatment (rIL-18). Data represent *n* = 7–15 mice/group as mean ± SEM from one (**d**–**g**) or two representative experiments (**a**–**c**). *P* values are determined by two-way ANOVA by Tukey’s multiple comparison analysis (**a**–**f**) and paired nonparametric Wilcoxon test (**g**). *p < 0.05; **p < 0.01; ***p < 0.001; ****p < 0.0001.

Since IL-18 has been previously shown to ameliorate severity of DSS colitis,^44,47,48^ we aimed to determine whether IL-18 supplementation would be sufficient to reduce inflammation in *Prevotella*-colonized mice. SPF and SPF + P mice were administered daily with recombinant IL-18 (rIL-18) or vehicle intraperitoneally (i.p.) starting 2 days prior and during the DSS colitis. Indeed, administration of rIL-18 attenuated colitis severity in mice colonized with *P. intestinalis*, as assessed by reduced weight loss (Fig. 5a, b) and histological examination of colon sections performed on day 7 post DSS induction (Fig. 5c, d). While both SPF mice groups, receiving PBS and rIL-18, showed similar mild crypt erosion, *Prevotella*-colonized mice administered with rIL-18, but not PBS, showed diminished colitis severity. *Prevotella*-colonized mice injected with PBS displayed more severe epithelial hyperplasia and mucosal invasion of inflammatory cells in comparison to mice supplemented with rIL-18 (SPF + P + rIL-18) (Fig. 5d). While administration of rIL-18 to SPF + P mice moderately reduced the levels of IL-6 and TNF-α (Fig. 5e), levels of chemoattractants MCP-1, MIP-1a, MIP-1b, and LIX were significantly diminished in comparison to PBS treated mice (Fig. 5f). Notably, supplementation of rIL-18 did not alter the abundance of *P. intestinalis* (Fig. 5g). Together, these results demonstrate that *Prevotella*-induced suppression of colonic IL-18 production alters susceptibility to intestinal inflammation upon tissue damage.

### Reduction of IL-18 is associated with *Prevotella*-induced changes in the microbiota and modulation of SCFAs production

**Fig. 6:**
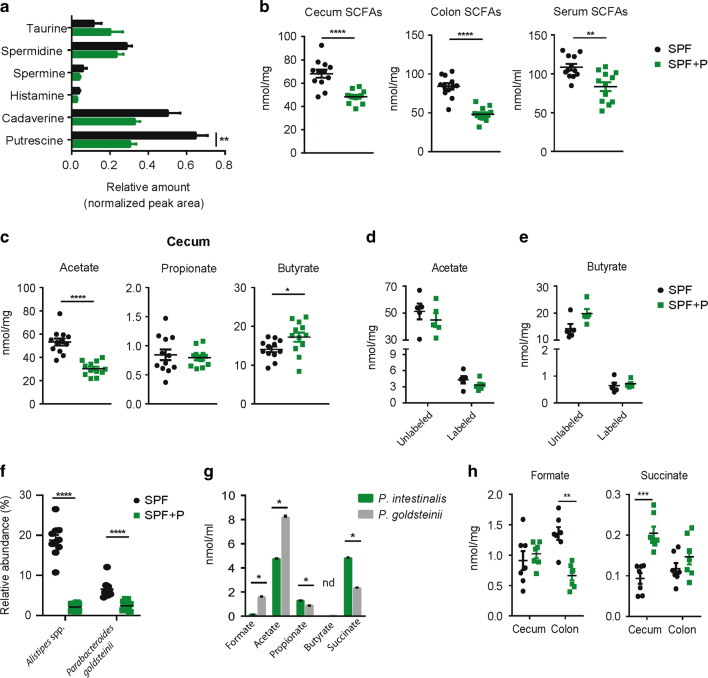
*Prevotella* colonization modulates SCFAs production. **a** Relative concentration of metabolites from cecal content of SPF and SPF + P mice analyzed by GC-MS in selective ion monitoring (SIM) mode. **b** Concentration of SCFAs (sum of acetate, propionate, and butyrate) in cecum, colon and serum. **c** Concentration of acetate, propionate, and butyrate in cecum content of SPF and SPF + P mice after 4 weeks of colonization, analyzed by GC-MS. **d**, **e** Concentration of ^13^C-labeled and unlabeled acetate and butyrate in cecum content of SPF and SPF + P mice 4 h after oral gavage of 50 mg of ^13^C-labeled acetate. **f** Relative abundance of present Bacteroidetes OTUs in SPF and SPF + P mice. **g** Concentration of fermentation products in *P. intestinalis* and *P. parabacteroides* cultures in BHI-S medium at OD600 = 0.45. **h** Concentration of formate and succinate in cecum and colon content of SPF and SPF + P mice. Data represent *n* = 5–12 mice/group as mean ± SEM from one (**d**, **e**) or two representative experiments (**a**–**c**, **f**). *P* values indicated represent an unpaired nonparametric Mann–Whitney test. *p < 0.05; **p < 0.01; ***p < 0.001; ****p < 0.0001.

Distinct microbial metabolites, specifically taurine, histamine, polyamines, and SCFAs modulate inflammasome signaling on the transcriptional and post-transcriptional level.^47,48^ Hence, we first addressed whether changes in IL-18 protein levels observed in *Prevotella*-colonized mice were accompanied by changes on the transcriptional level. Indeed, *P. intestinalis* colonization resulted in reduced *Il18* expression, while *Casp1* expression was not significantly affected (Supplementary Fig. 5a). While taurine has been demonstrated to enhance IL-18 processing via activation of the Nlrp6 inflammasome, histamine and distinct polyamines have been shown to have an inhibitory effect.^47^ Therefore, we measured taurine, histamine, putrescine, spermine, spermidine, and cadaverine concentrations in cecal content of SPF and SPF + P mice, however, we did not observe any correlation between the relative amounts of detected metabolites and levels of IL-18 (Fig. 6a). Conversely, mice with SPF microbiota, which showed higher levels of colonic IL-18, displayed a twofold increase in luminal putrescine concentrations than the *Prevotella*-colonized mice (Fig. 6a). Also, antimicrobial peptides previously identified to be modulated by IL-18 in the colon were not altered (Supplementary Fig. 5b).^47^

The role of SCFA in the maintenance of epithelial health has been extensively investigated.^48–52^ Mackay and colleagues demonstrated that the SCFAs acetate and butyrate can act on GPR43 and GPR109a receptors on IECs, respectively, and stimulate the expression of the *Il18* gene in the intestine.^48^ Since we recently showed modulation of SCFA levels 8 weeks after *Prevotella* spp. colonization resulting in alterations of osteoclast metabolism in the bone,^53^ we hypothesized that *P. intestinalis* also induced a decrease in SCFAs earlier after colonization, thereby resulting in distinct IL-18 production in SPF and SPF + P mice. Measurements of the concentrations of SCFAs in the intestinal content and serum of SPF and SPF + P mice 4 weeks after *Prevotella* colonization revealed that total SCFAs levels were affected already at this time point (Fig. 6b). More specifically, we observed a decrease of SCFA concentration in the cecum and colon content by 30% and 43%, respectively, as well as in the serum in SPF + P mice (23% decrease in SPF + P mice) (Fig. 6b). The decrease mainly derived from a significant reduction of acetate concentrations, the most abundant SCFA, in cecum (−50%), colon (−35%), and serum (−25%) (Fig. 6c, Supplementary Fig. 5c, d). In contrast, propionate was slightly decreased only in the colon (Supplementary Fig. 5c), while butyrate showed minor opposing site-specific concentrations changes (Fig. 6c, Supplementary Fig. 5c, d). A similar reduction of colonic SCFA and acetate as well as concomitantly colonic IL-18 concentrations was observed in SPF2 + P mice (Supplementary Fig. 5e, f). These results show that *Prevotella* colonization modulates SCFAs production and suggest that the lower concentration of IL-18 in *Prevotella*-colonized mice is associated with changes in the concentration of acetate, but not other types of SCFA.

Next, we investigated how *P. intestinalis* colonization modulates the concentration of acetate and considered different mechanisms, i.e., the conversion in other SCFAs^54,55^ or distinct SCFA production profiles between resident commensals and *Prevotella*.^56^ First, although frequently regarded as an end product of anaerobic fermentation, acetate is utilized by specific intestinal bacteria for butyrate production.^55^ As a significant decrease of acetate and a concurrent increase of butyrate is observed after *P. intestinalis* colonization in the cecum, we sought to investigate whether *P. intestinalis* utilizes acetate for butyrate production. Therefore, *P. intestinalis* was cultured in the presence or absence of physiological concentrations of acetate. Notably, *P. intestinalis* did not produce any butyrate (Supplementary Fig. 5g) and acetate concentrations did not decrease when added exogenously. In the absence of added acetate, *P. intestinalis* produced acetate, propionate and succinate in similar concentrations as reported for *P. copri*.^57^ Next, we wanted to exclude that in vivo *P. intestinalis* convert acetate itself or promotes its conversion by other bacteria reducing its concentration. Therefore, SPF and SPF + P mice were given orally 50 mg of stable isotope (^13^C)-labeled acetate and SCFA were analyzed for ^13^C-enrichment after 4 h. The presence of bacteria producing butyrate from acetate within the SPF microbiota was detected in general as evidenced by the presence of ^13^C-labeled butyrate, however, we did not observe higher concentrations of ^13^C-labeled butyrate in SPF + P mice (Fig. 6d, e). Hence, we considered the presence of distinct fermentation profiles in *P. intestinalis* as the basis for reduced acetate concentration. Comparison of the production of metabolites after in vitro cultivation of *P. intestinalis* to *Parabacteroides goldsteinii*, one of the Bacteroidales representatives who was outcompeted by *P. intestinalis* (Fig. 6f), identified as fermentation products formate and acetate, as well as succinate and to a certain degree also propionate. Notably, *P. intestinalis* produced relatively more succinate and less acetate than *P. goldsteinii* (Fig. 6g). Related changes in the concentrations in succinate were observed in vivo, i.e., higher in SPF + P mice in the cecum (Fig. 6h). Of note, these changes were not observed in the colon (Fig. 6h).

To investigate whether these alterations in SCFA production are specific to *P. intestinalis*, SPF mice were colonized with another *Prevotella* species (new isolate, proposed name: *Prevotella rodentium*) (Gálvez, Iljazovic, manuscript in preparation) or a member of the family Muribaculaceae (phylum Bacteroidetes), i.e., *Duncaniella muris*,^58^ which is also absent in SPF mice. Microbiota analysis showed both strains were able to colonize SPF reaching abundances similar to *P. intestinalis* reducing the abundance of resident Bacteroidetes species (Supplementary Fig. 6a). Measurement of luminal SCFA concentrations revealed reductions in acetate comparable to *P. intestinalis* (Supplementary Fig. 6b, c). Concurrently, decrease of colonic IL-18 was measured upon *P. rodentium* and *D. muris* colonization (Supplementary Fig. 6d). Finally, colonization with *D. muris* enhanced susceptibility to DSS-induced intestinal inflammation in SPF mice, while a similar trend was observed for *P. rodentium* (Supplementary Fig. 6d) suggesting that the decrease of acetate production as an indirect result of domination by specific groups of Bacteroidales is more generally linked to enhanced inflammation after intestinal injury.

## Discussion

Numerous studies in humans and animal models have established associations between alteration in the microbiota composition and a wide range of inflammatory diseases, e.g., IBD and RA.^59,60^ Specifically, the increased relative abundances of members of Prevotellaceae family within diverse microbial ecosystems have been associated with RA,^22,61^ periodontitis,^62^ and intestinal and vaginal dysbiosis,^21,63–65^ yet the direct functional relevance of increased *Prevotella* colonization is largely unclear.

Some of the known *Prevotella* species have been reported to be involved in opportunistic infections, while most of them are classically considered to be commensals colonizing different mucosal sites.^39^ In the intestine, the presence of *Prevotella* spp. has been proposed to be a biomarker of one of the three human gut enterotypes in developed countries.^66^ Besides its association to RA in humans,^22,61^ some studies have observed changes in *Prevotella* abundances in IBD patients,^67,68^ whereas other studies showed no association.^9^ Conversely, members of the genus *Prevotella* have also been associated with beneficial effects on the health as well, such as improved glucose metabolism^25^ and correlation with plant-rich diet.^23,24^ Hence, whether the association of different host responses to *Prevotella* spp. colonization in human individuals is explained by high species diversity and different functional capabilities or by indirect community-mediated effects is not known.^69,70^ Finally, *Prevotella* may not be causatively involved and rather be a marker of a distinct state of the microbiome promoting inflammation. Altogether, these observations highlight the interest to expand our understanding of the impact of *Prevotella* spp. on the intestinal ecosystem and the host.

The lack of intestinal *Prevotella* isolates from experimental models prevents addressing these highly relevant questions. Specifically, as the gut microbiota of conventionally raised mice is often not permissive to colonization by human enteropathogens^71,72^ or commensal human-derived bacteria.^73,74^ Hence, commonly repetitive, daily gavage or antibiotics are used to overcome the colonization resistance, as in case of studies with *P. copri*.^22,25^ Antibiotics use in such models can have profound effects on the host physiology directly or via antibiotic-induced dysbiosis, making these models ambiguous. Therefore, we developed a targeted isolation strategy to recover novel *Prevotella* species from the mouse intestine and characterized the outcome of *P. intestinalis* colonization on a resident community and intestinal health. *P.* *intestinalis* colonized the mice in high relative abundance, similar to what has been reported for *P. copri* in humans,^70^ occupying the niche of resident bacteria in the colonic lumen and mucosa. The microbiota of recipient mice in this study was characterized by the high abundance of Firmicutes that have been in general associated with beneficial effects on the host.^8,59^ In turn, microbiota composition of *Prevotella*-colonized mice was characterized by a decrease in α-diversity (Shannon) and members of the Firmicutes phylum, both changes previously attributed to dysbiosis and microbiota of IBD, asthma, and RA patients.^75–77^ Colonization by *P. intestinalis* did not lead to intestinal inflammation in immunocompetent mice without inducing injury to the intestine (up to 5 weeks of colonization). Yet, upon induction of damage to the intestinal barrier and exposure to the luminal bacteria in the DSS colitis model, *Prevotella*-colonized mice displayed signs of exacerbated inflammation in comparison to SPF mice. Importantly, exacerbation of inflammation was not restricted to the mice with one specific gut microbiome composition used in this study (SPF), as comparable results were recapitulated in commercially obtained mice with a distinct microbiome composition (SPF2). Specifically, the inflamed tissue of mice colonized with *P. intestinalis* was characterized by increased levels of IL-6 and TNF-α, as well as higher levels of neutrophil-attracting chemokines accompanied with neutrophils infiltration. Strikingly, while the intestinal community of Nlrp6-deficient mice, from which *P. intestinalis* was isolated, exacerbates DSS colitis in a T-cell dependent manner,^32^ *P. intestinalis* did not require adaptive immune cells to exacerbate disease, i.e., Rag2-deficient mice showed *Prevotella*-exacerbated intestinal inflammation. These results suggest that other members or combined effects of distinct microbes in the colitogenic community in *Nlrp6*^*−/−*^ mice are responsible for induction of pro-inflammatory adaptive immune cells. Subsequent analysis clearly demonstrated that *Prevotella* colonization shapes host immunity already during the steady state, i.e., reduction of IL-18 production. Specifically, we observed that *P. intestinalis* colonization of SPF microbiota results in significant decrease of colonic *Il18* expression and IL-18 production (2.5-fold) during steady state. Interestingly, previous studies demonstrated that decrease of IL-18 production by 1.3–1.5-fold was already sufficient to disturb the intestinal homeostasis.^47,48^ Hence, we hypothesized that these changes affected the intestinal barrier during steady state, which in turn contributed to a more severe intestinal inflammation in *Prevotella*-colonized mice during DSS colitis. Indeed, rIL-18 supplementation ameliorated the susceptibility to intestinal inflammation in *Prevotella*-colonized mice and alleviated colonic tissue damage. Notably, even though IL-18 has been widely studied, no definitive role of IL-18 in intestinal homeostasis and inflammation has been conclusively established. While some studies suggested IL-18 has a protective role, preventing dysbiosis^21,47^ and promoting epithelial barrier integrity and regeneration,^44,48^ others have linked IL-18 to increased colitis severity.^45,46^ Our data further add to the complex role of IL-18 in the intestine during homeostasis and inflammation suggesting that perturbation of the levels of IL-18 levels may predispose to intestinal inflammation upon tissue injury.

Several microbiota-derived metabolites in the intestine have been shown to modulate IL-18 production, either by effecting *Il18* expression^49,78^ or through modulation of inflammasome activation,^47,48^ e.g., SCFAs, taurine and distinct polyamines such as spermidine. Targeted metabolomics revealed that colonization of the SPF community by *P. intestinalis* is accompanied by a significant decrease in SCFAs levels, in particular, acetate, but none of the other known Nlrp6 inflammasome modulators. As an end product of microbial fermentation, intestinal production of SCFAs, specifically straight-chain SCFAs, are influenced by both diet and microbiota composition. While production of butyrate has been associated to Clostridia, members of the Bacteroidetes have been reported to be the major contributor to propionate and acetate production.^56,79^ Surprisingly, *Prevotella*-induced microbiota changes in SPF mice resulted in a decrease, rather than the expected increase, of acetate concentration in the intestine of SPF + P mice. We initially hypothesized *P. intestinalis* may be directly utilizing acetate for butyrate production as this has been reported for other gut commensals, however, our data showed that *P. intestinalis* produced formate, acetate, propionate, and succinate, while no butyrate production was detected in vitro. Experiments with stable isotope-labeled acetate showed that *Prevotella* does not utilize acetate for butyrate production itself, or stimulates the conversion in other bacteria in vivo as no significant increase of ^13^C-labeled butyrate in SPF + P mice was detected. As an alternative to an enhanced consumption of acetate, we considered that *Prevotella*-induced changes in the microbiota reduce acetate production. Indeed, *P. goldsteinii*, which was strongly reduced by *Prevotella* and is one of the two members of the Bacteroidetes in SPF mice, produced relatively more acetate and formate, while *P. intestinalis* fermentation resulted in higher levels of propionate. In turn, succinate, an alternative fermentation product, was produced at higher levels by *P. intestinalis* in vitro supporting the distinct metabolism of *P. intestinalis* compared to other microbiota members. Of note, succinate was not consistently changed in vivo being increased in the cecum but not colon of SPF + P in comparison to SPF mice. As succinate is a major fermentation product of *P. copri*^80^ further research will be needed to evaluate the importance of changes in succinate and other metabolites produced by *Prevotella* spp. of the mouse and human intestinal microbiota to host biology. Specifically, we conclude that *P. intestinalis* may be a valuable model organism for future studies of the association between *P. copri* and diverse diseases including RA enabling detailed longitudinal analysis of diverse physiological parameters and aspects of immune responses in vivo. It remains to be addressed which metabolic and associated changes in the host are specific for *Prevotella* spp. compared with other Bacteroidales groups. While colonization with a representative member of the family Muribaculaceae, which is highly prevalent in mice, exacerbates disease similarly to *P. intestinalis*, notable additive effects were observed after colonization of SPF2 mice with *P. intestinalis*. Despite the presence of Muribaculaceae and another *Prevotella* spp. in SPF2 mice, colonization with *P. intestinalis* further exacerbated disease severity likely reflecting its ability to efficiently colonize the mouse gut

Taken together, our data provide strong evidence for an immunomodulatory role of *Prevotella* spp. in the intestine. The reduction of SCFA concentrations in SPF + P mice is linked to a distinct fermentation profile in *P. intestinalis* combined with a relative reduction of predominantly acetate-producing bacteria due *to P. intestinalis* expansion. Down-modulation of microbial acetate production after intestinal domination by specific groups of Bacteroidales may more general result in an enhancement of intestinal inflammation after chemical damage to the intestinal barrier.

## Materials and methods

### Mice

Wild-type (WT), *Rag2*^*−/−*^, and IL-17A^GFP^ IFN-γ^Katushka^ FoxP3^RFP^ reporter mice used in the study were on the C57BL/6N background. They were all bred and maintained at the animal facilities of the Helmholtz Centre for Infection Research (HZI) under enhanced specific pathogen-free conditions (SPF) (Table S3). Therefore, mice were rederived into SPF microbiota by embryo transfer.^30^ C57BL/6N “SPF2” mice were obtained from a commercial vendor (Taconic) (Table S4). *Nlrp6*^*−/−*^ mice were obtained from Yale University and maintained under conventional housing conditions at the HZI without rederivation. All experiments were performed with 10- to 11-week-old age-matched and gender-matched animals.

### *P. intestinalis* isolation and colonization of mice

Fresh colonic content of conventionally housed donor *Nlrp6*^*−/−*^ mice was collected in BBL thioglycollate media and fecal content homogenate was further processed in an anaerobic chamber with following gas mixture: 70% nitrogen, 20% carbon dioxide, and 10% hydrogen. Bacteria were isolated by using the most probable number (MPN) technique (Goodman et al.^81^) where homogenized content was diluted in a range in which maximal 30% of wells showed detectable growth. Specifically, 10-fold dilutions (10^−6^ and 10^−7^) of fecal content homogenate were cultured in a sterile 96-well plate in Brain Hearth Infusion broth supplemented with 10% FBS and 0.5 g/L vitamin K3 (BHI-S medium) on 37 °C for 2 days. *Prevotella*-positive wells were further enriched in BHI-S medium containing vancomycin (6 µg/ml) and selected on BHI-S blood agar (defibrinated sheep blood, 5% v/v) plates. Positive colonies were passaged three times on agar plates before a pure culture was obtained and glycerol stocks were frozen in −80 °C.

For every experiment, *P. intestinalis, P. rodentium*, or *Duncaniella muris* culture was grown anaerobically (70% N_2_, 20% CO_2_, and 10% H_2_) from a frozen glycerol stock in BHI-S medium on 37 °C for 2 days. All mice were colonized by oral gavage at age of 5–6 weeks with each bacteria at a dose 3 × 10^8^ CFU in 200 µl of BHI media. With exception, SPF2 mice were colonized with *P. intestinalis* at the same dose by oral gavage, followed by a 20–30 µl rectal administration.

### DSS-induced colitis

Acute colitis was induced by adding dextran sodium sulfate (DSS) in sterilized drinking water of 10–11-week-old WT and gene-deficient mice. Mice with SPF and SPF2 microbiome composition were given 2.1% (w/v) and 1.6% (w/v) DSS in drinking water, respectively, for 7 days, followed by 5 days of access to regular drinking water. During the course of DSS treatment fresh DSS solution was prepared and replaced on day 0 and day 4. Mice were monitored daily by measurement of body weight and clinical assessment, including stool consistency and detection of blood in the stool. Animals which lost 20% or more of their initial body weight were euthanized.

### Statistical analysis

Statistical analysis was performed using GraphPad Prism 7 program (GraphPad Software, Inc.) and R v3.3.0. Data are expressed as mean ± SEM (Standard error of mean). Differences were analyzed by Student’s *t* test and ANOVA. *P* values indicated represent an unpaired nonparametric Mann–Whitney or two-way ANOVA by Tukey’s multiple comparison analysis. The permutational multivariate ANOVA analysis of variance (ADONIS) was computed with 999 permutations. For ADONIS tests, an *R*^2^ > 0.1 (effect size, 10%) and *p* value < 0.05 were considered as significant. *P* values ≤ 0.05 were considered as significant: *p < 0.05, **p < 0.01, ***p < 0.001, ****p < 0.0001.

## Supplementary information

Supplementary Information

Supplementary Fig. 1

Supplementary Fig. 2

Supplementary Fig. 3

Supplementary Fig. 4

Supplementary Fig. 5

Supplementary Fig. 6

Supplementary Table 1

Supplementary Table 2

Supplementary Table 3

Supplementary Table 4

## References

[1] Belkaid Y, Harrison OJ (2017). Homeostatic immunity and the microbiota. Immunity.

[2] Peterson LW, Artis D (2014). Intestinal epithelial cells: regulators of barrier function and immune homeostasis. Nat. Rev. Immunol..

[3] Postler TS, Ghosh S (2017). Understanding the holobiont: how microbial metabolites affect human health and shape the immune system. Cell Metab..

[4] Swidsinski A (2002). Mucosal flora in inflammatory bowel disease. Gastroenterology.

[5] Sartor RB (2006). Mechanisms of Disease: pathogenesis of Crohn’s disease and ulcerative colitis. Nat. Clin. Pract. Gastroenterol. Hepatol..

[6] Ni J, Wu GD, Albenberg L, Tomov VT (2017). Gut microbiota and IBD: causation or correlation?. Nat. Rev. Gastroenterol. Hepatol..

[7] Tedelind S, Westberg F, Kjerrulf M, Vidal A (2007). Anti-inflammatory properties of the short-chain fatty acids acetate and propionate: a study with relevance to inflammatory bowel disease. World J. Gastroenterol..

[8] Manichanh C (2006). Reduced diversity of faecal microbiota in Crohn’s disease revealed by a metagenomic approach. Gut.

[9] Morgan XC (2012). Dysfunction of the intestinal microbiome in inflammatory bowel disease and treatment. Genome Biol..

[10] Makki K, Deehan EC, Walter J, Bäckhed F (2018). The impact of dietary fiber on gut microbiota in host health and disease. Cell Host Microbe.

[11] Darfeuille-Michaud A (2004). High prevalence of adherent-invasive *Escherichia coli* associated with ileal mucosa in Crohn’s disease. Gastroenterology.

[12] Carvalho FA (2012). Transient inability to manage proteobacteria promotes chronic gut inflammation in TLR5-deficient mice. Cell Host Microbe.

[13] Carvalho FA (2009). Crohn’s disease adherent-invasive *Escherichia coli* colonize and induce strong gut inflammation in transgenic mice expressing human CEACAM. J. Exp. Med..

[14] Garrett WS (2010). Enterobacteriaceae act in concert with the gut microbiota to induce spontaneous and maternally transmitted colitis. Cell Host Microbe.

[15] Seo S-U (2015). Distinct commensals induce interleukin-1β via NLRP3 inflammasome in inflammatory monocytes to promote intestinal inflammation in response to injury. Immunity.

[16] Seregin SS (2017). NLRP6 protects Il10−/− mice from colitis by limiting colonization of *Akkermansia muciniphila*. Cell Rep..

[17] Bloom SM (2011). Commensal bacteroides species induce colitis in host-genotype-specific fashion in a mouse model of inflammatory Bowel disease. Cell Host Microbe.

[18] Kullberg MC (1998). Helicobacter hepaticus triggers colitis in specific-pathogen-free interleukin-10 (IL-10)-deficient mice through an IL-12- and gamma interferon-dependent mechanism. Infect. Immun..

[19] Escalante NK (2016). The common mouse protozoa Tritrichomonas muris alters mucosal T cell homeostasis and colitis susceptibility. J. Exp. Med..

[20] Fernandes MA (2019). Enteric virome and bacterial microbiota in children with ulcerative colitis and Crohn disease. J. Pediatr. Gastroenterol. Nutr..

[21] Elinav E (2011). NLRP6 inflammasome regulates colonic microbial ecology and risk for colitis. Cell.

[22] Scher, J. U. et al. Expansion of intestinal *Prevotella copri* correlates with enhanced susceptibility to arthritis. *Elife* **2**, e01202 (2013). 10.7554/eLife.01202 PMC3816614 24192039

[23] Clemente JC (2015). The microbiome of uncontacted Amerindians. Sci. Adv..

[24] Martínez I (2015). The gut microbiota of rural papua new guineans: composition, diversity patterns, and ecological processes. Cell Rep..

[25] Kovatcheva-Datchary P (2015). Dietary fiber-induced improvement in glucose metabolism is associated with increased abundance of prevotella. Cell Metab..

[26] Pedersen HK (2016). Human gut microbes impact host serum metabolome and insulin sensitivity. Nature.

[27] Leite AZ (2017). Detection of increased plasma interleukin-6 levels and prevalence of *Prevotella copri* and *Bacteroides vulgatus* in the feces of type 2 diabetes patients. Front. Immunol..

[28] Rodriguez, D. A. et al. *Prevotella copri* in individuals at risk for rheumatoid arthritis. 1–4 10.1136/annrheumdis-2018-214514 (2019).

[29] Palm NW (2014). Immunoglobulin A coating identifies colitogenic bacteria in inflammatory Bowel Disease. Cell.

[30] Gálvez, E. J. C., Iljazovic, A., Gronow, A., Flavell, R. & Strowig, T. Shaping of intestinal microbiota in Nlrp6- and Rag2-deficient mice depends on community structure. *Cell Rep*. **21**, 3914–3926 (2017). 10.1016/j.celrep.2017.12.027 29281837

[31] Mamantopoulos M (2017). Nlrp6- and ASC-dependent inflammasomes do not shape the commensal gut microbiota composition. Immunity.

[32] Roy U (2017). Distinct microbial communities trigger colitis development upon intestinal barrier damage via innate or adaptive immune cells. Cell Rep..

[33] Lesker TR (2020). An integrated metagenome catalog reveals new insights into the murine gut microbiome. Cell Rep..

[34] Gu S (2013). Bacterial community mapping of the mouse gastrointestinal tract. PLoS ONE.

[35] Yasuda K (2015). Biogeography of the intestinal mucosal and lumenal microbiome in the rhesus macaque. Cell Host Microbe.

[36] Wang A-H (2016). Human colorectal mucosal microbiota correlates with its host niche physiology revealed by endomicroscopy. Sci. Rep..

[37] Rolhion N (2019). A *Listeria monocytogenes* bacteriocin can target the commensal *Prevotella copri* and modulate intestinal infection. Cell Host Microbe.

[38] Vandeputte D (2017). Quantitative microbiome profiling links gut community variation to microbial load. Nature.

[39] Larsen JM (2017). The immune response to Prevotella bacteria in chronic inflammatory disease. Immunology.

[40] Macpherson AJ, McCoy KD (2015). Standardised animal models of host microbial mutualism. Mucosal Immunol..

[41] Chassaing, B., Aitken, J. D., Malleshappa, M. & Vijay-Kumar, M. in *Current Protocols in Immunology* Vol. 104, 15.25.1–15.25.14 (John Wiley & Sons, Inc., 2014). 10.1002/0471142735.im1525s104 PMC3980572 24510619

[42] Oficjalska K (2015). Protective role for caspase-11 during acute experimental murine colitis. J. Immunol..

[43] Salcedo R (2010). MyD88-mediated signaling prevents development of adenocarcinomas of the colon: role of interleukin 18. J. Exp. Med..

[44] Zaki MH (2010). The NLRP3 inflammasome protects against loss of epithelial integrity and mortality during experimental colitis. Immunity.

[45] Błażejewski AJ (2017). Microbiota normalization reveals that canonical caspase-1 activation exacerbates chemically induced intestinal inflammation. Cell Rep..

[46] Nowarski R (2015). Epithelial IL-18 equilibrium controls barrier function in colitis. Cell.

[47] Levy M (2015). Microbiota-modulated metabolites shape the intestinal microenvironment by regulating NLRP6 inflammasome signaling. Cell.

[48] Macia L (2015). Metabolite-sensing receptors GPR43 and GPR109A facilitate dietary fibre-induced gut homeostasis through regulation of the inflammasome. Nat. Commun..

[49] Singh N (2014). Activation of Gpr109a, receptor for niacin and the commensal metabolite butyrate, suppresses colonic inflammation and carcinogenesis. Immunity.

[50] Smith PM (2013). The microbial metabolites, short-chain fatty acids, regulate colonic treg cell homeostasis. Science.

[51] Park J (2015). Short-chain fatty acids induce both effector and regulatory T cells by suppression of histone deacetylases and regulation of the mTOR–S6K pathway. Mucosal Immunol..

[52] Furusawa Y (2013). Commensal microbe-derived butyrate induces the differentiation of colonic regulatory T cells. Nature.

[53] Lucas S (2018). Short-chain fatty acids regulate systemic bone mass and protect from pathological bone loss. Nat. Commun..

[54] den Besten G (2014). The short-chain fatty acid uptake fluxes by mice on a guar gum supplemented diet associate with amelioration of major biomarkers of the metabolic syndrome. PLoS ONE.

[55] Duncan SH, Barcenilla A, Stewart CS, Pryde SE, Flint HJ (2002). Acetate utilization and butyryl coenzyme A (CoA):acetate-CoA transferase in butyrate-producing bacteria from the human large intestine. Appl. Environ. Microbiol..

[56] Flint HJ, Duncan SH, Scott KP, Louis P (2015). Links between diet, gut microbiota composition and gut metabolism. Proc. Nutr. Soc..

[57] Franke T, Deppenmeier U (2018). Physiology and central carbon metabolism of the gut bacterium *Prevotella copri*. Mol. Microbiol..

[58] Lagkouvardos, I. et al. Sequence and cultivation study of Muribaculaceae reveals novel species, host preference, and functional potential of this yet undescribed family. *Microbiome* **7**, 28 (2019). 10.1186/s40168-019-0637-2 PMC6381624 30782206

[59] Frank DN (2007). Molecular-phylogenetic characterization of microbial community imbalances in human inflammatory bowel diseases. Proc. Natl Acad. Sci. USA.

[60] Zhang X (2015). The oral and gut microbiomes are perturbed in rheumatoid arthritis and partly normalized after treatment. Nat. Med..

[61] de Aquino SG (2014). Periodontal pathogens directly promote autoimmune experimental arthritis by inducing a TLR2- and IL-1-driven Th17 response. J. Immunol..

[62] Ji S, Kim Y, Min B-M, Han SH, Choi Y (2007). Innate immune responses of gingival epithelial cells to nonperiodontopathic and periodontopathic bacteria. J. Periodontal Res..

[63] Gosmann C (2017). Lactobacillus-deficient cervicovaginal bacterial communities are associated with increased HIV acquisition in young south african women. Immunity.

[64] Onderdonk AB, Delaney ML, Fichorova RN (2016). The human microbiome during bacterial vaginosis. Clin. Microbiol. Rev..

[65] Dillon SM (2016). Gut dendritic cell activation links an altered colonic microbiome to mucosal and systemic T-cell activation in untreated HIV-1 infection. Mucosal Immunol..

[66] Arumugam M (2011). Enterotypes of the human gut microbiome. Nature.

[67] Lucke K (2006). Prevalence of *Bacteroides* and *Prevotella* spp. in ulcerative colitis. J. Med. Microbiol..

[68] Kleessen B, Kroesen AJ, Buhr HJ, Blaut M (2002). Mucosal and invading bacteria in patients with inflammatory bowel disease compared with controls. Scand. J. Gastroenterol..

[69] Kumar Gupta, V., Chaudhari, N. M., Iskepalli, S. & Dutta, C. Divergences in gene repertoire among the reference Prevotella genomes derived from distinct body sites of human. 10.1186/s12864-015-1350-6 (2011). 10.1186/s12864-015-1350-6 PMC4359502 25887946

[70] De Filippis F (2019). Distinct genetic and functional traits of human intestinal *Prevotella copri* strains are associated with different habitual diets. Cell Host Microbe.

[71] Barthel M (2003). Pretreatment of mice with streptomycin provides a *Salmonella enterica* serovar Typhimurium colitis model that allows analysis of both pathogen and host. Infect. Immun..

[72] Ritchie, J. M. in *Enterohemorrhagic Escherichia coli and Other Shiga Toxin-Producing E. coli* Vol. 2, 175–195 (American Society of Microbiology, 2014).

[73] Chung H (2012). Gut immune maturation depends on colonization with a host-specific microbiota. Cell.

[74] Lee SM (2013). Bacterial colonization factors control specificity and stability of the gut microbiota. Nature.

[75] Wright EK (2015). Recent advances in characterizing the gastrointestinal microbiome in Crohn’s disease: a systematic review. Inflamm. Bowel Dis..

[76] Abrahamsson TR (2014). Low gut microbiota diversity in early infancy precedes asthma at school age. Clin. Exp. Allergy.

[77] Maeda Y (2016). Dysbiosis contributes to arthritis development via activation of autoreactive T cells in the intestine. Arthritis Rheumatol..

[78] Kalina U (2002). Enhanced production of IL-18 in butyrate-treated intestinal epithelium by stimulation of the proximal promoter region. Eur. J. Immunol..

[79] den Besten G (2013). The role of short-chain fatty acids in the interplay between diet, gut microbiota, and host energy metabolism. J. Lipid Res..

[80] De Vadder F (2016). Microbiota-produced succinate improves glucose homeostasis via intestinal gluconeogenesis. Cell Metab..

[81] Goodman AL (2011). Extensive personal human gut microbiota culture collections characterized and manipulated in gnotobiotic mice. Proc Natl Acad Sci U S A.

