# Supplementary material for: Perturbation of the gut microbiome by Prevotella spp. enhances host susceptibility to mucosal inflammation
Source: Mucosal Immunol. 2020 May 20;14(1):113–24. doi: 10.1038/s41385-020-0296-4 (PMC7790746; doi:10.1038/s41385-020-0296-4)
Supplement: Supplementary file 1 — Supplementary Information [file 41385_2020_296_MOESM1_ESM.docx]

**Supplementary information**

**Fig. S1: Intestinal colonization by *P. intestinalis***

**a** Analysis of α-diversity (Observed and Shannon) in SPF and SPF+P mice (Student t-test). **b** Gating strategy and **c** quantification of flow cytometric enumeration of microbial cell concentrations in luminal content (cecum and colon) of SPF and SPF+P mice. **d** Relative abundance of *P. intestinalis* in the lumen and mucosa of DC and PC colon. **e** Quantitative PCR based determination of *P. intestinalis* abundance during steady state in stool, colon mucosa and tissue, mLN, and liver.

Results represent n=5-18 mice/group as mean ± SEM from at least two independent experiments. P values indicated represent unpaired nonparametric Mann-Whitney test unless stated otherwise *p < 0.05; **p < 0.01; ***p< 0.001; ****p< 0.0001

**Fig. S2: *P. intestinalis* colonization exacerbates colonic inflammation in DSS-colitis independent of the resident microbiota**

**a** Survival of WT mice harboring SPF and SPF+P microbiota during DSS colitis (2.1 % for 7 days). **b** Histological evaluation of H&E stained cecum and **c** small intestine sections on d7 after DSS induction. **d** Relative abundance of *P. intestinalis* in feces of SPF+P mice on d0 and d6 of DSS quantified by 16s rRNA sequencing, and **e** Quantitative PCR based determination of *P. intestinalis* abundance during DSS (d7) in colon mucosa and tissue, mLN, and liver. **f** Fecal microbiota composition analysis of SPF2 and SPF2+P WT mice on the family level and **g** relative abundance of *Prevotella* OTUs present in nontreated SPF2 and SPF2+P, using 16S rRNA gene sequencing. **h** Body weight loss and **i** survival of WT mice harboring SPF2 and SPF2+P microbiota during DSS colitis (1.6 % w/v for 7 days). **j** Colon length and **e** histological evaluation of SPF2 and SPF2+P mice evaluated during DSS colitis (d7).

Data represent n=6-12 mice/group as mean ± SEM from one out of three representative experiments. P values indicated represent an unpaired nonparametric Mann-Whitney test *p < 0.05; **p < 0.01; ***p< 0.001; ****p< 0.0001.

**Fig. S3: Analysis of distinct pro-inflammatory cytokines in SPF and SPF+P mice during steady state and DSS-colitis**

**a** Cytokines and chemokines measured from distal colon tissue homogenates of SPF and SPF+P mice during steady state (d0) and on day 7 of DSS, analyzed using a LEGENDplex kit. Data represent n=4-8 mice/group as mean ± SEM from one out of two representative experiments. P values indicated represent an unpaired nonparametric Mann-Whitney *p < 0.05.

**Fig. S4: Global analysis of colonic LPLs identifies innate cells as a major contributor to intestinal inflammation in *Prevotell*a-colonized mice**

**a-f** Colonic lamina propria leukocytes (cLPLs) were isolated from WT mice harboring SPF and SPF+P microbiota, during the steady state (d0) and on day 7 (d7) during DSS colitis, and analyzed by fluorescence-activated cell sorting (FACS). **a** Gating strategy of FACS data is displayed for subsets of helper T cells and innate cells (**d)**. **b** Frequency of CD45+ cells in cLPLs. **c** Total numbers of distinct helper t cell subsets at d0 and d7 of DSS. **e** frequency of neutrophils during d0 and d7 of DSS and **f** total cell numbers of distinct innate immune cells on d7 of DSS. **g** Colon length of WT and Rag2-deficient mice untreated or colonized with *P. intestinalis* on d7 of DSS colitis.

Data represent n=5-10 mice/group as mean ± SEM from one out of three independent experiments. P values indicated represent an unpaired nonparametric Mann-Whitney test *p < 0.05; **p < 0.01; ***p< 0.001; ****p< 0.0001.

**Fig. S5: Changes in SCFAs concentration are associated with altered *Il18* gene expression**

**a** Colonic *Casp1* and *Il18* and **b** antimicrobial peptides *(Ang4, Retnlβ, RegIIIγ*) gene expression in mice with SPF and SPF+P microbiota, 4-5 weeks after colonization. **c-d** Concentration of acetate, propionate, and butyrate in colonic content and serum of SPF and SPF+P mice after 4 weeks of colonization, analyzed by GC-MS. **e** concentration of SCFA and acetate measured from colon content and **f** colonic IL-18 levels of SPF2 and SPF2+P mice after 4 weeks of colonization **g** Time-resolved analysis of the concentration of fermentation products by *P. intestinalis* cultured in BHI-S (BHI) and BHI-S supplemented with 50 mM acetate (BHI+acetate) (n=3).

Data represent n=11-15 mice/group (A-C) as mean ± SEM from two representative experiments. P values indicated represent an unpaired nonparametric Mann-Whitney test *p < 0.05; **p < 0.01; ***p< 0.001; ****p< 0.0001.

**Fig. S6: *P. rodentium* and *D. muris* modulate intestinal microbiota and metabolite production similar to *P. intestinalis***

**a-d** Fecal microbiota composition, concentration of **b** SCFA and **c** acetate measured in colonic content, and **d** colonic IL-18 concentration of SPF mice, untreated or colonized for 4 weeks with *P. rodentium* or *D. muris*. **e** Body weight loss of SPF mice, untreated or colonized with *P. rodentium* or *D. muris,* during DSS colitis (2.1 % w/v for 7 days).

Data represent n=10-12 mice/group as mean ± SEM from two representative experiments. P values indicated represent an unpaired nonparametric Mann-Whitney test *p < 0.05; **p < 0.01; ***p< 0.001; ****p< 0.0001.

**Supplementary experimental procedures**

**DNA isolation and 16S rRNA gene-based microbial community analysis**

Fresh stool samples or luminal content were collected from mice and immediately stored at

-20^o^C. DNA was extracted using phenol/chloroform based purification as previously described^1^. In brief, a samples were suspended in a solution containing 500 μL of extraction buffer (200 mM Tris, 20 mM EDTA, and 200 mM NaCl [pH 8.0]), 200 μL of 20% SDS, 500 μL of phenol:chloroform:isoamyl alcohol (24:24:1), together with 0.1 mM zirconia/silica beads and homogenized twice using bead beater (BioSpec) for 2 min. After precipitation of DNA, crude DNA extracts were resuspended in Tris-EDTA (TE) buffer with 100 μg/mL RNase and column-purified to remove PCR inhibitors (BioBasic).

For DNA isolation from mucosa-associated bacteria, a previously reported protocol has been adapted^2^. Briefly, collected colons were opened longitudinally and content was removed by washing two times in sterile 1xPBS, or until all visible content was removed. Mucus associated bacteria were detached from the intestinal wall in three 1-min washes in 12.5 ml 0.1% (w/v) Tween 80 in 1xPBS by vigorously shaking the tube. The washes were pooled and centrifuged at 10,000 rpm, for 10 min at 4°C to pellet the cells. DNA from bacterial pellets was further isolated as described for stool samples.

Amplification of the V4 region (F515/R806) of the 16S rRNA gene was performed as previously described protocols^3^. Samples were sequenced on an Illumina MiSeq platform (PE250). Barcode-based demultiplexing was performed using IDEMP software with default parameters (https://github.com/yhwu/idemp). Obtained reads were assembled, quality controlled and clustered using Usearch8.1 software package (http://www.drive5.com/usearch/). Briefly, reads were merged using -fastq_mergepairs –with fastq_maxdiffs 30 and quality filtering was done with fastq_filter (-fastq_maxee 1), minimum read length 200 bp. The OTU clusters and representative sequences were determined using the UPARSE algorithm^4^, followed by taxonomy assignment using the Silva database v128^5^ and the RDP Classifier^6^ with a bootstrap confidence cutoff of 80% performed by using QIIME v1.8.0^7^. OTU absolute abundance table and mapping file were used for statistical analyses and data visualization in the R statistical programming environment package phyloseq^8^. To determine bacterial OTUs that explained differences between microbiota settings, the LEfSe method was used^9^. OTUs with Kruskal-Wallis test < 0.05 and LDA scores > 4.0 were considered informative.

**Recovering *P. intestinalis* prevalence in iMGMC**

The genome of the *P. intestinalis* was clustered together with all mMAGs (n=1296) of the iMGMC resource^10^ with dRep^11^ using a Mash-ANI of 95%. *P. intestinalis* was assigned to one cluster with the representative genome extra-SRR6945393.5. The corresponding iMGMC genome cluster (59_0) contain 51 MAGs reconstructed from samples of 6 different studies listed in Table S2.

***P. intestinalis* quantification using quantitative PCR**

To measure the relative abundances of *P. intestinalis* in mice stool, mucosa, mLN, and liver, DNA extraction was performed as described in previous section, and RT-PCR was performed using 5 ng/μl template DNA, primers pairs specific for 16S rRNA gene (F: ACTCCTACGGGAGGCAGCAGT, R: ATTACCGCGGCTGCTGGC) and *P. intestinalis* (F: CGTCCCTTGACGGCATCCGACA, R: CAGCCCCGAAGGGAAGGGGTG), and Kapa Probe Syber qPCR kit (Kapa Biosystems) on a LightCycler 480 instrument (Roche). PCR conditions were 95°C for 60 s, followed by 40 cycles of amplification (95°C for 3 s, 60°C for 30 s). Data were analyzed using the deltaCt method with 16S rRNA cp values serving as a reference.

**Bacterial cell enumeration by flow cytometry**

Colon and cecum content from SPF and SPF+P mice was suspended in 10 ml BHI medium, weighed, and filtered through 70 µM cell strainer. 0.5 mg/ml of filtered stool suspension was centrifuged for 10 min at 5,000 rpm, and the bacterial pellet was washed twice in 1x PBS. 1x10^5^ of 6 µm fluorophore-containing beads was added to 200 μl of bacterial suspension, and flow cytometry analysis was performed using a BD LSR (BD Biosciences). Data were analyzed with FlowJo software (TreeStar Inc.). Number of bacteria was determined from the ratio of bacterial events to bead events.

**Colonoscopy**

Colonoscopy was performed on anesthetized mice using a high-resolution mouse video endoscopic system (‘Coloview’, Carl Storz, Tuttlingen, Germany). The severity of colitis was scored in a blinded manner using Murine Endoscopic Index of Colitis Severity (MEICS), which is based on five parameters: granularity of mucosal surface (0-3); vascular pattern (0-3); translucency of the colon mucosa (0-3); visible fibrin (0-3); and stool consistency (0-3)^12^.

**Histology**

Intestinal tissue samples were placed (cecum) or rolled up to “swiss roles” (colon and small intestine) in histology cassettes, fixed in 4% neutrally buffered formaldehyde for a maximum of 48 h, and stored in 70% ethanol until processing. Samples were further embedded in paraffin according to standard histological procedures and sections of 3 µm thickness were stained with hematoxylin-eosin (HE). Stained sections were evaluated under a light microscopy in a blind manner. Evaluation of the severity of colitis in DSS treated mice was done using adapted TJL-scoring system, developed by The Jackson Laboratory^13^. Adaptation of the score was previously described^14^. The colon was divided into a proximal (oral), middle and distal (aboral) section, each of about the same size. The three sections were scored for the general criteria: severity (0-3), ulceration (0-3), oedema (0-3), hyperplasia (0-3), inflammatory cell infiltration (0-3), and area involved (0-3) where score 0 depicted no alteration to score 3 massive alteration in the given parameters. The scores were added up to a total of up to 18 per section and the scores of the three sections to a total of up to 54 per colon sample.

**Determination of Host Gene and Protein Expression in Colon Tissue**

Colons were excised, washed in PBS, and divided into proximal and distal colon. Three centimeters of DC was cut longitudinally into two samples: one for RNA and other for protein extraction. RNA isolation was performed using TRI Reagent (Sigma-Aldrich) according to the manufacturer’s instructions. One microgram of RNA was used for cDNA synthesis (RevertAid Reverse Transcriptase, Thermo Fisher Scientific) using polyd(T)12-18 primer (Roth). Real-time PCR was done using the Kapa Probe Fast qPCR kit (Kapa Biosystems) and gene-specific probe sets (Il18 Mm_00434225_m1, Casp1 Mm_00438023_m1, Ang4 Mm_03647554_g1), Retnlb Mm_00445845_m1), and Reg3g Mm_0044127_m1) [Applied Biosystems]; Hprt [F: CTGGTGAAAAGGACCTCTCG; R: TGAAGTACTCATTATAGTCAAGGGCA; probe: TGTTGGATACAGGCCAGACTTTGTTGGAT]) on a LightCycler 480 instrument (Roche). PCR conditions were 95°C for 60 s, followed by 40 cycles of 95°C for 3 s and 60°C for 30 s. Data were analyzed using the deltaCt method, with Hprt serving as the reference housekeeping gene.

Protein extraction was performed by mechanical homogenization of DC tissue samples in NP-40 buffer, containing protease inhibitors (cOmplete Mini EDTA-free, Roche), using Mini-Beadbeater-96 (BioSpec). Tissue homogenates were further centrifuged (10,000 rpm for 5 min at 4°C), and the supernatants were collected for cytokine and chemokine measurements. All cytokines and chemokines were measured using LEGENDplex assays (Biolegend) according to the manufacturer’s instructions, while IL-18 was measured using the IL-18 ELISA kit (MBL International).

**Isolation of colonic lamina propria leukocytes (cLPL) and flow cytometry**

cLPL we isolated by density gradient centrifugation using Percoll as previously described (Nagano et al., 2012). In brief, colons were collected during steady state and at d7 of DSS treatment. Colons were opened longitudinally, washed with PBS and then shaken in HBSS buffer containing 2 mM EDTA for 20 min at 37°C. Tissue was cut into small pieces and incubated for 20 min at 37°C in the digestion solution (DMEM containing 1% fetal bovine serum (FBS), 0.25 mg/ml collagenase D, 0.5 U/ml dispase and 5 µg/ml DNase I) while shaking. Digested tissues were filtered through 70µM cell strainer (Falcon) and DMEM + 5% FBS was added to inactivate enzymes. The last two steps were repeated until all tissue was digested. For the innate cells analysis, cells were centrifuged and suspended in staining buffer containing PBS, 1% FBS and 2 mM EDTA, while Percoll gradient was performed for further separation of cells of adaptive immune cells. In brief, cells were suspended in 4 ml of 40% Percoll (GE Healthcare) and overlaid on 4 ml of 80% Percoll. Gradient separation was performed by centrifugation at 450 g for 25 min at 25°C. Cells in the interphase were collected and suspended in staining buffer containing PBS, 1% FBS and 2 mM EDTA. The following antibodies were used: anti-CD45 (30-F11), anti-CD3 (17A2), anti-CD4 (RM4-5, GK1.5), anti-CD8a (53-6.7), anti-CD44 (IM7), anti-CD62L (MEL-14), anti-MHC class II (M5/114.15.2), anti-CD11b (M1/70), anti-CD11c (N418), anti-Ly6G (IA8), anti-Ly6C (HK1.4), anti-F4/80 (BM8) (Biolegend), anti-CCR2 (SA203G11), anti-CX3CR1 (SA011F11). To distinguish live dead cells AlexaFluor-350 NHS Ester (Life Technologies) was used. Flow cytometry analysis was performed using a BD LSR (BD Biosciences) and data were analyzed with FlowJo software (TreeStar Inc.).

**Metabolite measurements and *in vivo* stable isotope labeling**

Cecum and colon content samples were weighted, snap-frozen in liquid nitrogen and stored at -80°C until further processing. For SCFAs extraction fecal content (50-100 mg) was resuspended in 600 µl water spiked with internal standard (2 µl o-cresol/250 ml) and 60 µl 65% HPLC-grade sulfuric acid per 50 mg fresh weight and mixed vigorously for 5 min. 400 µl of the mixture were extracted with 200 µl of tert-butyl methyl ether. For culture supernatants, volatile (except formate) and non-volatile fermentation products were extracted as described previously^15^. Acetate, propionate and butyrate^15^ as well as succinate^16^ were analyzed as described previously. Formate was derivatized and analyzed as described by Tumanov et al.^17^ with minor modifications: The column was a Agilent HP-5ms Ultra Inert (30 m x 0.25 mm x 0.25 µm) and the GC oven temperature was hold for 1 min at 60 °C and increased to 150 °C at a rate of 12 °C/min followed by an increase to 300 °C with a rate of 50 °C/min. Standard curves of formate, acetate, propionate, butyrate and succinate were extracted and analyzed the same way and were used for external calibration.

For *in vivo* stable isotope labeling experiment, each mouse was gavaged with 50 mg of ^13^C-acetate dissolved in 0.2 ml NaCl solution (0.9%). Cecal content was collected 4h after oral gavage. The mass isotopomer distribution was determined based on the non-derivatized molecular ion of the SCFA and corrected for the natural occurrence of 13C according to the number of carbon atoms in the molecule.

For extraction of taurine, histamine, and polyamines the protocol was adapted from ^18^. Briefly, cecal content (50-100 mg) was collected in tubes containing glass and ceramic beads mix (Bertin-corp) and appropriate volume (1000 µL/100 mg cecal content) of -20 °C ice-cold extraction fluid 1 (methanol+H_2_O, 4+1) containing 32 µM U^13^C ribitol as internal standard was added. Homogenization was performed with a Retsch mill (3x 2 min at 30 Hz/sec). An appropriate volume (500 µL/100 mg cecal content) of extraction fluid 2 (H_2_O containing 2 µg/mL D^6^-glutaric acid) was added and samples were mixed for 10 sec. Chloroform (800 µL/100 mg cecal content) was added and samples were again mixed for 30 sec prior agitation for 15 min at 1400 rpm and 4 °C in a tube shaker. After centrifugation for 5 min at 4 °C and 13.000 rpm, 60 µL of the upper polar phase was transferred to a GC vial with micro insert. Solvents were evaporated at 4 °C in a rotary vacuum evaporator until total dryness. Sample tubes were capped and stored at -80 °C until further processing.

Online metabolite derivatization was performed using an Axel Semrau Autosampler. Dried polar metabolites were dissolved in 15 μL of 2% methoxyamine hydrochloride in pyridine at 40 °C under shaking. After 90 min, an equal volume of N-methyl-N-(trimethylsilyl)-trifluoracetamide (MSTFA) was added and held for 30 min at 40 °C.

Sample (1 µL) was injected into an SSL injector at 270 °C in splitless mode. GC-MS analysis was performed using an Agilent 7890A GC equipped with a J&W 30m DB-35MS capillary column (0.25 mm inner diameter, 0.25 µm film thickness). Helium was used as carrier gas at a flow rate of 1.0 mL/min. The GC oven temperature was held at 90 °C for 1 min and increased to 320 °C at a rate of 15 °C/min and held at that temperature for 8 min, resulting in a total run time of 25 min per sample. The GC was connected to an Agilent 5975C MS operating under electron impact ionization at 70 eV. The transfer line temperature was set to 280 °C. The MS source was held at 230 °C and the quadrupole at 150 °C. Tuning and maintenance of the GC-MS was done according to the supplier´s instructions, an automated tuning routine was applied every 150 injections. Data processing was done using the MetaboliteDetector software ^19^*.*

**rIL-18 mice treatment**

Each mouse received intraperitoneal (i.p.) injections of 200 ng of mouse recombinant IL-18 suspended in 200 µL sterile PBS for 8 consecutive days, starting 1 day before administration of DSS in drinking water. Control mice were administered daily with 200 µL of sterile PBS.

**Supplementary information references:**

1. Roy, U. *et al.* Distinct Microbial Communities Trigger Colitis Development upon Intestinal Barrier Damage via Innate or Adaptive Immune Cells. *Cell Rep.* **21**, 994–1008 (2017).

2. Gong, J. *et al.* Diversity and phylogenetic analysis of bacteria in the mucosa of chicken ceca and comparison with bacteria in the cecal lumen. *FEMS Microbiol. Lett.* **208**, 1–7 (2002).

3. Caporaso, J. G. *et al.* Global patterns of 16S rRNA diversity at a depth of millions of sequences per sample. *Proc. Natl. Acad. Sci.* **108**, 4516–4522 (2011).

4. Edgar, R. C. UPARSE: highly accurate OTU sequences from microbial amplicon reads. *Nat. Methods* **10**, 996–998 (2013).

5. Quast, C. *et al.* The SILVA ribosomal RNA gene database project: improved data processing and web-based tools. *Nucleic Acids Res.* **41**, D590-6 (2013).

6. Wang, Q., Garrity, G. M., Tiedje, J. M. & Cole, J. R. Naive Bayesian classifier for rapid assignment of rRNA sequences into the new bacterial taxonomy. *Appl. Environ. Microbiol.* **73**, 5261–7 (2007).

7. Caporaso, J. G. *et al.* QIIME allows analysis of high-throughput community sequencing data. *Nat. Methods* **7**, 335–6 (2010).

8. McMurdie, P. J. & Holmes, S. phyloseq: an R package for reproducible interactive analysis and graphics of microbiome census data. *PLoS One* **8**, e61217 (2013).

9. Segata, N. *et al.* Metagenomic biomarker discovery and explanation. *Genome Biol.* **12**, R60 (2011).

10. Lesker, T. R. *et al.* An Integrated Metagenome Catalog Reveals New Insights into the Murine Gut Microbiome. *Cell Rep.* **30**, 2909–2922.e6 (2020).

11. Olm, M. R., Brown, C. T., Brooks, B. & Banfield, J. F. DRep: A tool for fast and accurate genomic comparisons that enables improved genome recovery from metagenomes through de-replication. *ISME J.* **11**, 2864–2868 (2017).

12. Becker, C., Fantini, M. C. & Neurath, M. F. High resolution colonoscopy in live mice. *Nat. Protoc.* **1**, 2900–4 (2006).

13. Mähler, M. *et al.* Genetic Analysis of Susceptibility to Dextran Sulfate Sodium-Induced Colitis in Mice. *Genomics* **55**, 147–156 (1999).

14. Pils, M. C. *et al.* Monocytes/macrophages and/or neutrophils are the target of IL-10 in the LPS endotoxemia model. *Eur. J. Immunol.* **40**, 443–448 (2010).

15. Neumann-Schaal, M., Hofmann, J. D., Will, S. E. & Schomburg, D. Time-resolved amino acid uptake of Clostridium difficile 630Δerm and concomitant fermentation product and toxin formation. *BMC Microbiol.* **15**, 281 (2015).

16. Will, S. E. *et al.* Day and Night: Metabolic Profiles and Evolutionary Relationships of Six Axenic Non-Marine Cyanobacteria. *Genome Biol. Evol* **11**, 270–294 (2018).

17. Tumanov, S., Bulusu, V., Gottlieb, E. & Kamphorst, J. J. A rapid method for quantifying free and bound acetate based on alkylation and GC-MS analysis. *Cancer Metab.* **4**, 17 (2016).

18. Jäger, C., Hiller, K. & Buttini, M. Metabolic Profiling and Quantification of Neurotransmitters in Mouse Brain by Gas Chromatography-Mass Spectrometry. *Curr. Protoc. Mouse Biol.* **6**, 333–42 (2016).

19. Hiller, K. *et al.* MetaboliteDetector: comprehensive analysis tool for targeted and nontargeted GC/MS based metabolome analysis. *Anal. Chem.* **81**, 3429–39 (2009).
