# Supplementary figures and images for: Perturbation of the gut microbiome by Prevotella spp. enhances host susceptibility to mucosal inflammation
Source: Mucosal Immunol. 2020 May 20;14(1):113–24. doi: 10.1038/s41385-020-0296-4 (PMC7790746; doi:10.1038/s41385-020-0296-4)

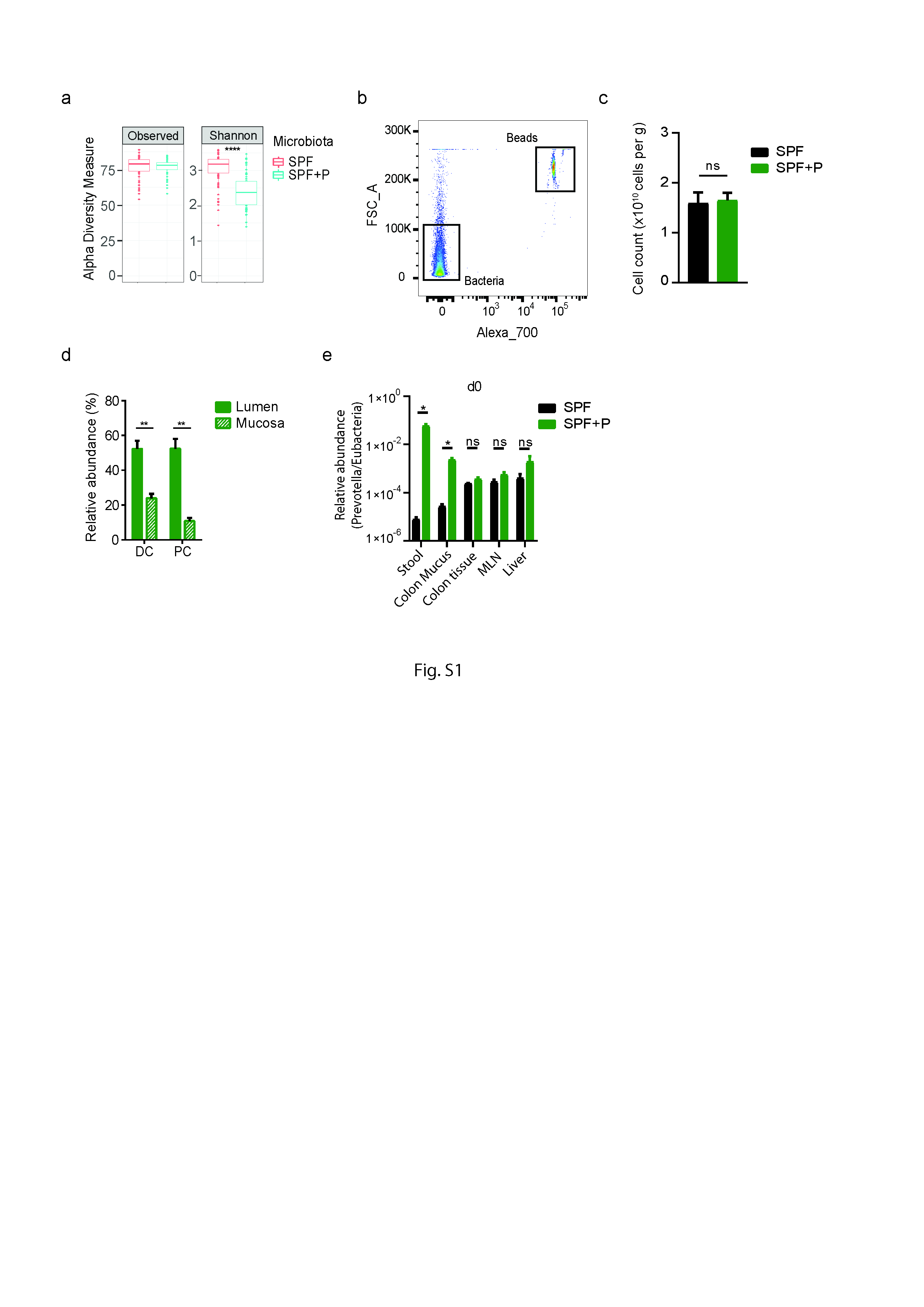

Supplement: Supplementary file 2 — Supplementary Fig. 1 [file 41385_2020_296_MOESM2_ESM.tif]

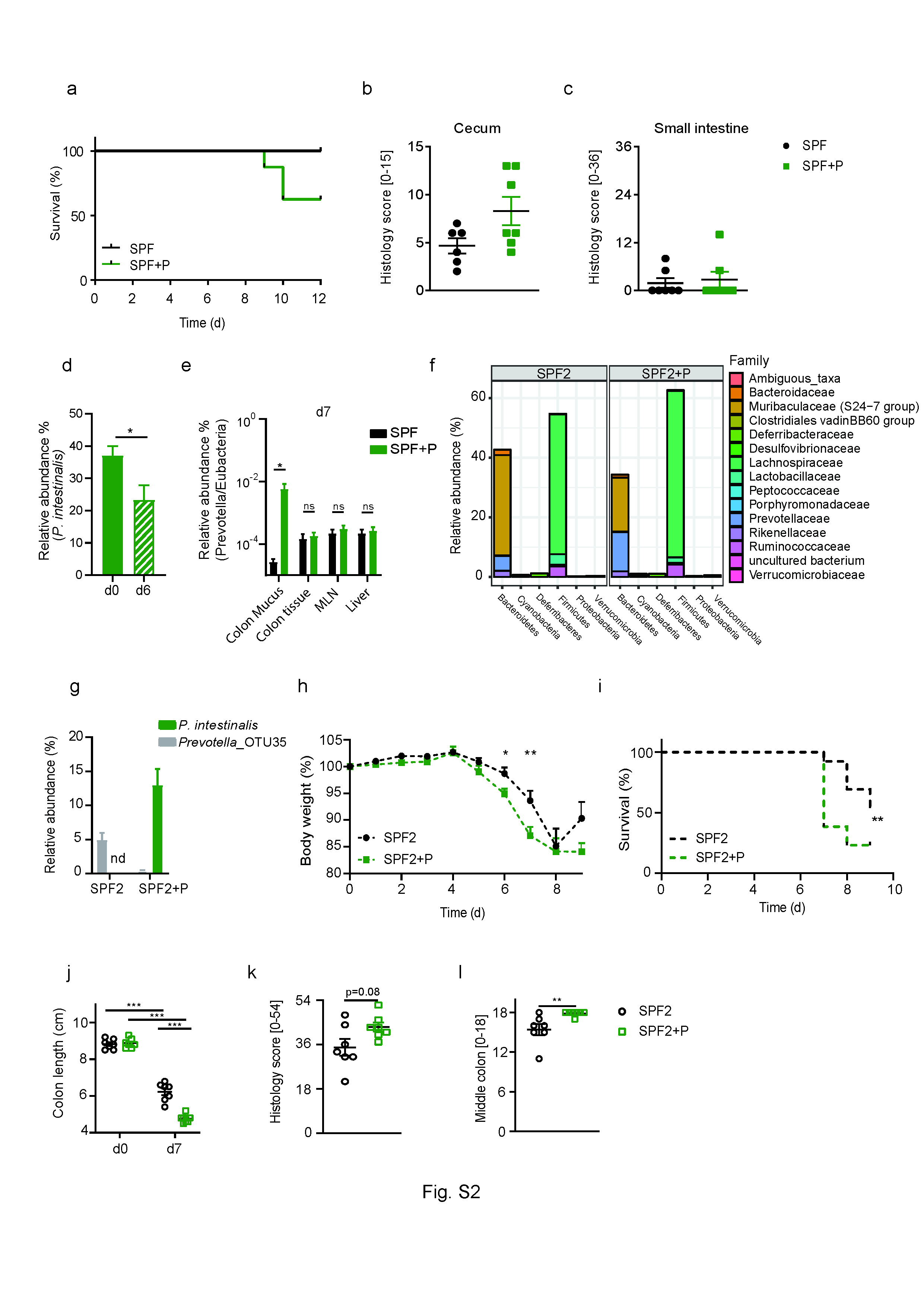

Supplement: Supplementary file 3 — Supplementary Fig. 2 [file 41385_2020_296_MOESM3_ESM.tif]

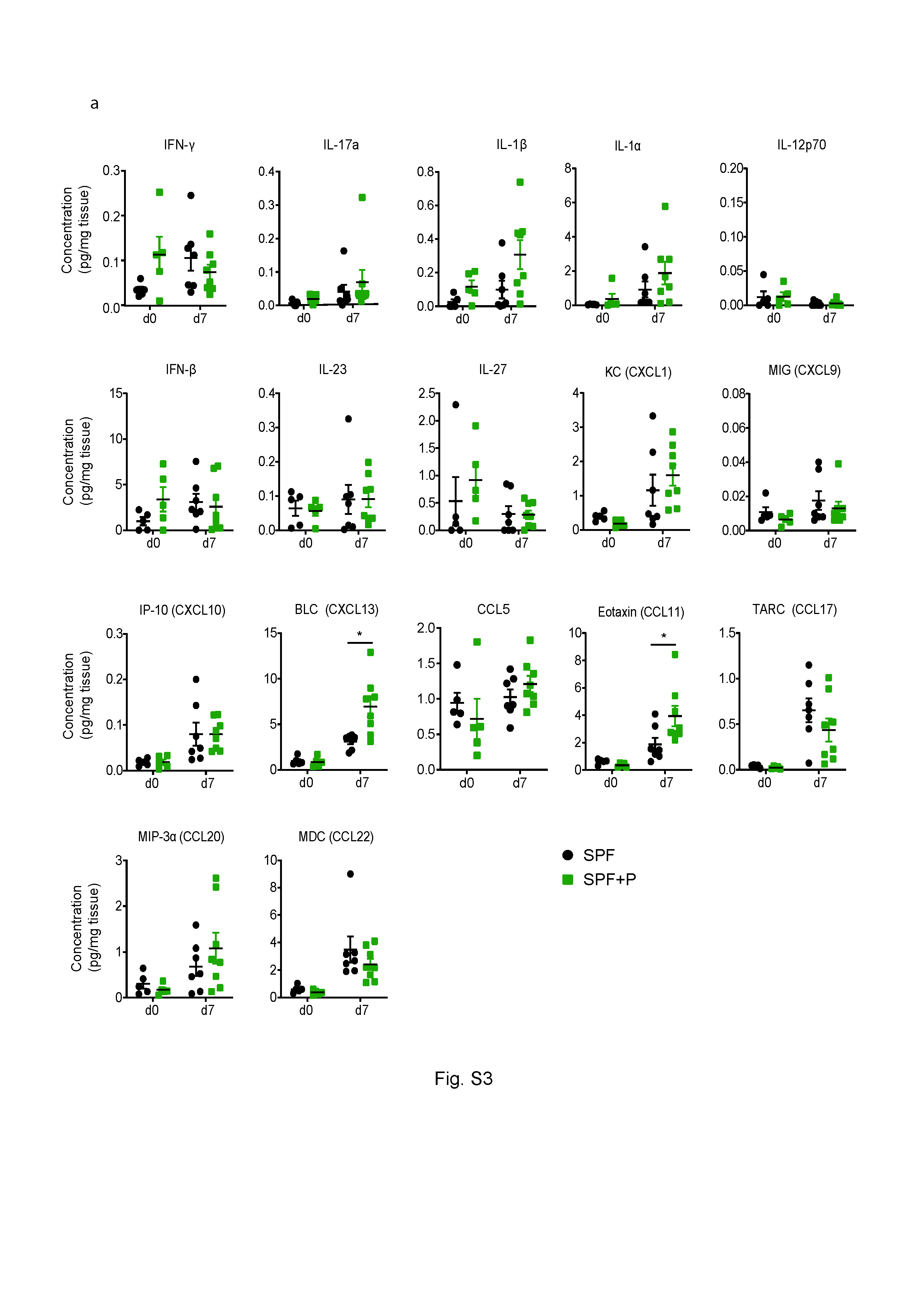

Supplement: Supplementary file 4 — Supplementary Fig. 3 [file 41385_2020_296_MOESM4_ESM.tif]

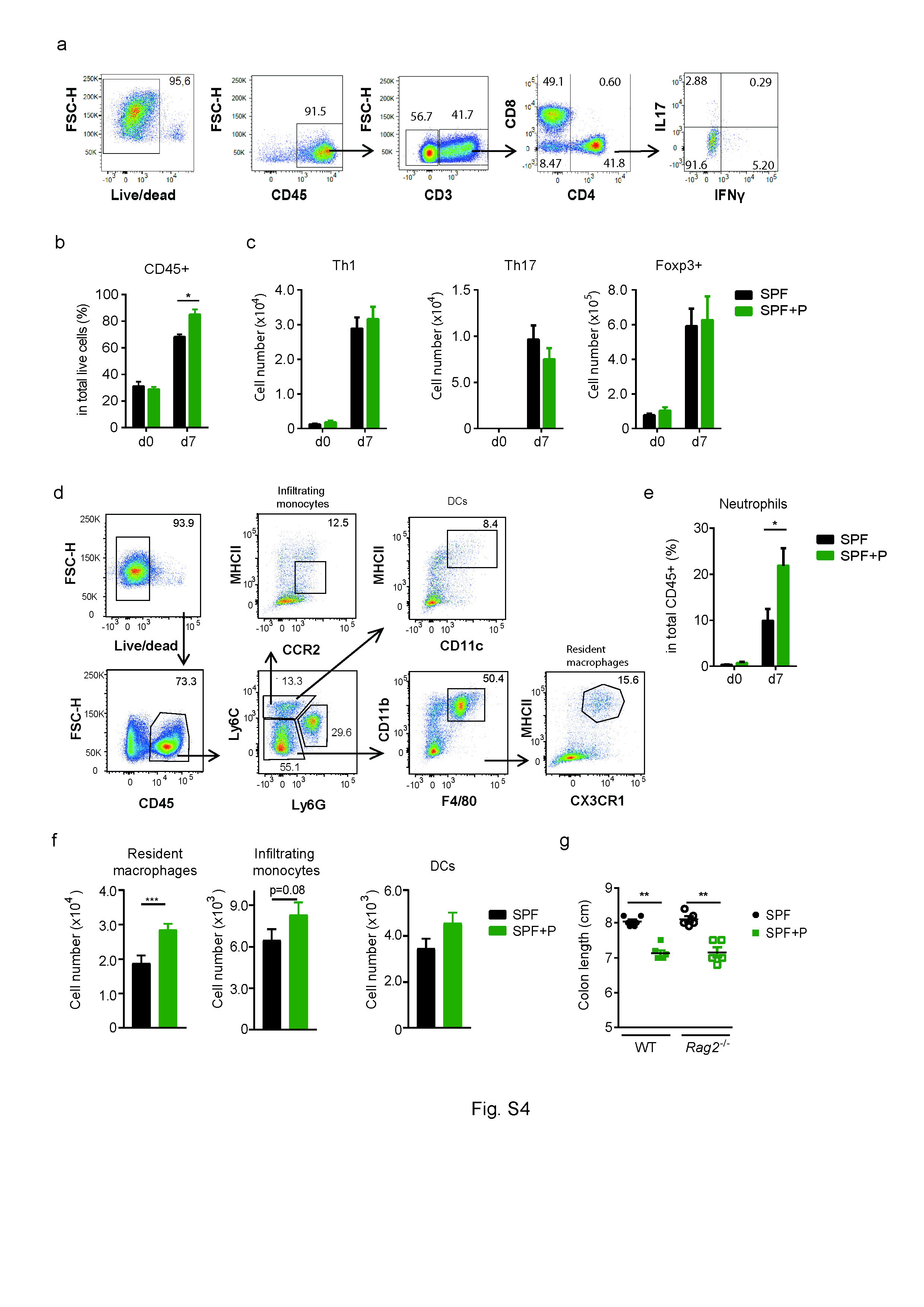

Supplement: Supplementary file 5 — Supplementary Fig. 4 [file 41385_2020_296_MOESM5_ESM.tif]

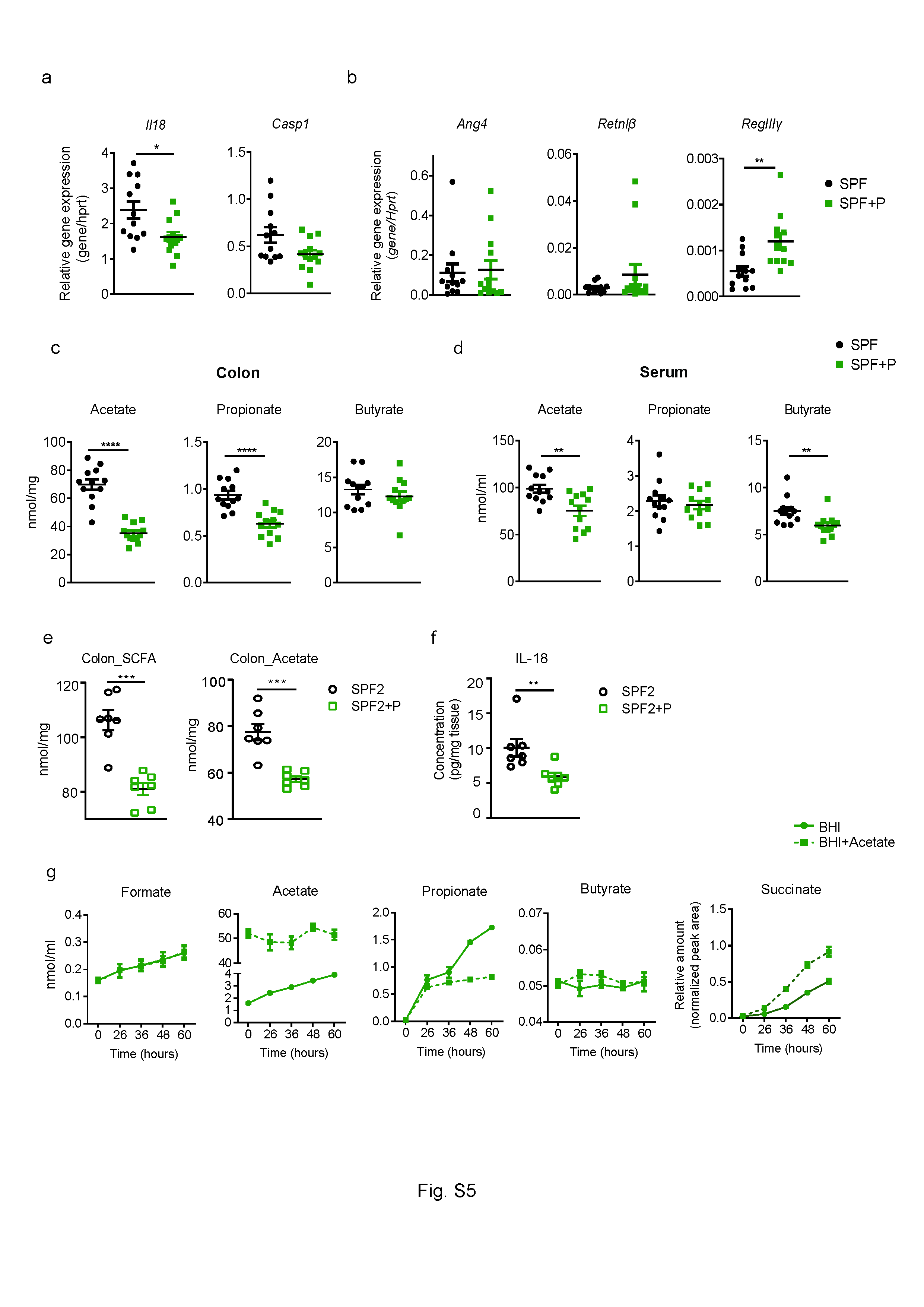

Supplement: Supplementary file 6 — Supplementary Fig. 5 [file 41385_2020_296_MOESM6_ESM.tif]

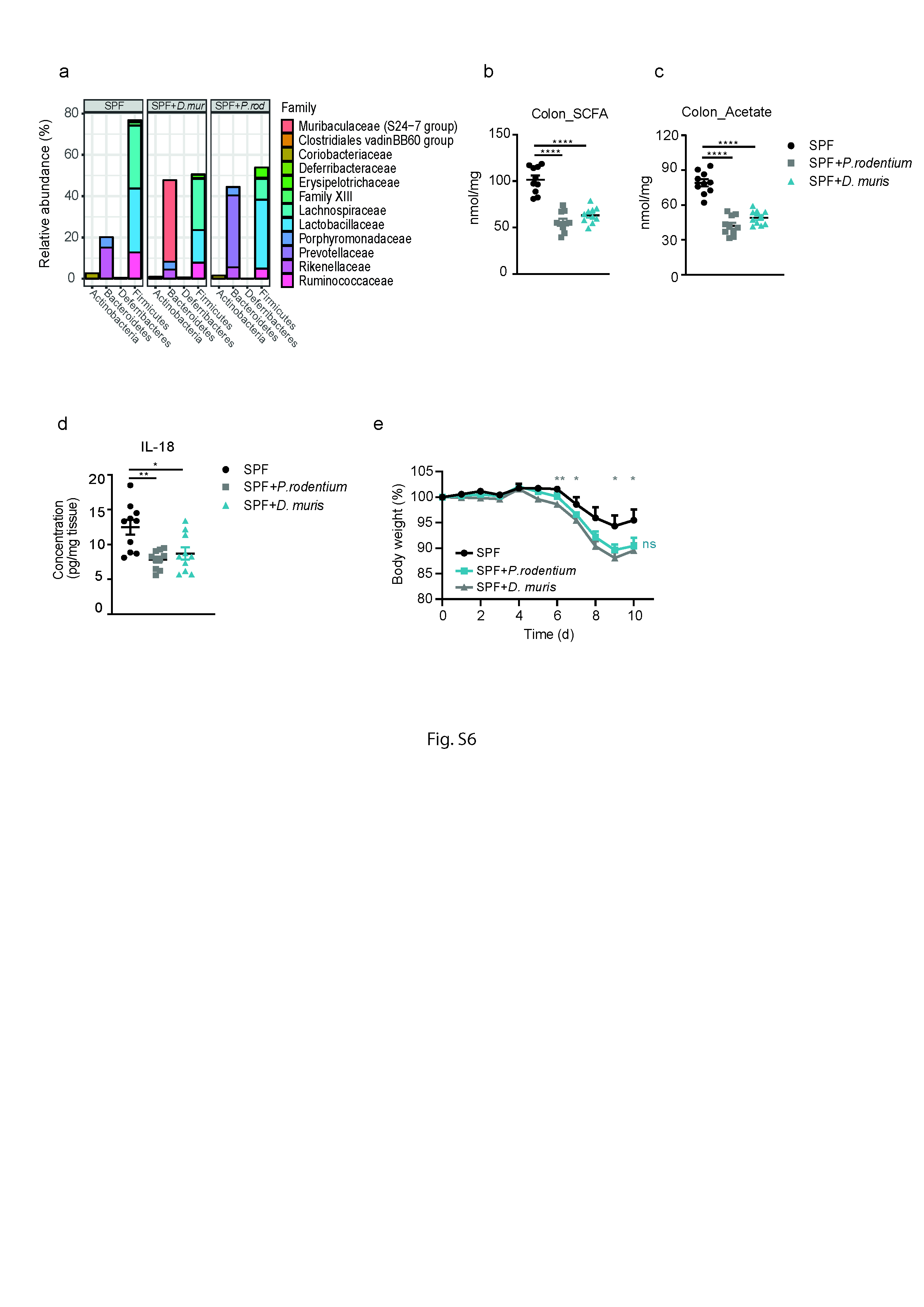

Supplement: Supplementary file 7 — Supplementary Fig. 6 [file 41385_2020_296_MOESM7_ESM.tif]
